# Evaluation of the Sensitivity of Metabolic Profiling by Rapid Evaporative Ionization Mass Spectrometry: Toward More Radical Oral Cavity Cancer Resections

**DOI:** 10.1021/acs.analchem.1c03583

**Published:** 2022-05-03

**Authors:** Pierre-Maxence Vaysse, Imke Demers, Mari F. C. M. van den Hout, Wouter van de Worp, Ian G. M. Anthony, Laura W. J. Baijens, Bing I. Tan, Martin Lacko, Lauretta A. A. Vaassen, Auke van Mierlo, Ramon C. J. Langen, Ernst-Jan M. Speel, Ron M. A. Heeren, Tiffany Porta Siegel, Bernd Kremer

**Affiliations:** † Maastricht MultiModal Molecular Imaging Institute (M4i), Division of Imaging Mass Spectrometry, Maastricht University, Universiteitssingel 50, 6229 ER Maastricht, The Netherlands; § Department of Otorhinolaryngology, Head and Neck Surgery, Maastricht University Medical Center, 6202 AZ Maastricht, The Netherlands; ∥ Department of Surgery, Maastricht University Medical Center, 6229 ER Maastricht, The Netherlands; ⊥ Department of Pathology, Maastricht University Medical Center, 6202 AZ Maastricht, The Netherlands; # GROW School for Oncology and Developmental Biology, Maastricht University Medical Center, 6202 AZ Maastricht, The Netherlands; ○ Department of Respiratory Medicine, NUTRIM School for Nutrition, Toxicology and Metabolism, Maastricht University Medical Center, 6202 AZ Maastricht, The Netherlands; □ Department of Cranio-Maxillofacial Surgery, Head and Neck Surgery, Maastricht University Medical Center, 6202 AZ Maastricht, The Netherlands

## Abstract

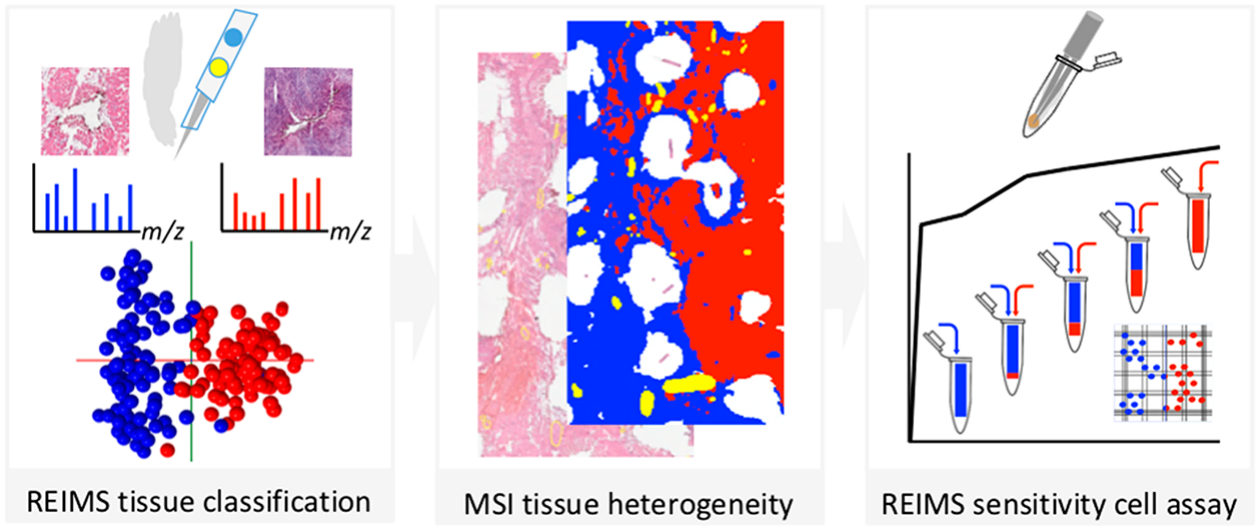

Radical resection for patients with oral cavity cancer remains challenging. Rapid evaporative ionization mass spectrometry (REIMS) of electrosurgical vapors has been reported for real-time classification of normal and tumor tissues for numerous surgical applications. However, the infiltrative pattern of invasion of oral squamous cell carcinomas (OSCC) challenges the ability of REIMS to detect low amounts of tumor cells. We evaluate REIMS sensitivity to determine the minimal amount of detected tumors cells during oral cavity cancer surgery. A total of 11 OSCC patients were included in this study. The tissue classification based on 185 REIMS *ex vivo* metabolic profiles from five patients was compared to histopathology classification using multivariate analysis and leave-one-patient-out cross-validation. Vapors were analyzed *in vivo* by REIMS during four glossectomies. Complementary desorption electrospray ionization–mass spectrometry imaging (DESI-MSI) was employed to map tissue heterogeneity on six oral cavity sections to support REIMS findings. REIMS sensitivity was assessed with a new cell-based assay consisting of mixtures of cell lines (tumor, myoblasts, keratinocytes). Our results depict REIMS classified tumor and soft tissues with 96.8% accuracy. *In vivo* REIMS generated intense mass spectrometric signals. REIMS detected 10% of tumor cells mixed with 90% myoblasts with 83% sensitivity and 82% specificity. DESI-MSI underlined distinct metabolic profiles of nerve features and a metabolic shift phosphatidylethanolamine PE(O-16:1/18:2))/cholesterol sulfate common to both mucosal maturation and OSCC differentiation. In conclusion, the assessment of tissue heterogeneity with DESI-MSI and REIMS sensitivity with cell mixtures characterized sensitive metabolic profiles toward *in vivo* tissue recognition during oral cavity cancer surgeries.

## Introduction

Surgery is the first choice treatment for patients with oral cavity cancer, including mobile tongue cancer.^1^ Surgeons aim for radical tumor extirpation with tumor-free resection margins resulting in the best possible disease-free survival^2^ and for optimal preservation of uninvolved tissues to maintain essential functions such as speaking and eating.^3^ However, the definitive radicality of the resection is assessed only after histological processing of the resection specimen, followed by meticulous microscopical assessment of the distance between tumor cells and the resection margins by a trained pathologist, which can take days to weeks before being reported. Therefore, intraoperative decisions remain challenging to reach radical tumor resection with preservation of function in patients with oral cavity cancer, despite possible assessments of the resection margin by frozen sections or other methods like Raman spectroscopy or narrow band imaging.^4,5^

Most oral cavity cancers are diagnosed as oral squamous cell carcinoma (OSCC). Next to the histopathological diagnosis, prognostic features like differentiation grade, perineural, lymphoangio-invasive growth, and pattern of invasion (POI), defined by the way tumor cells infiltrate into normal tissues, are assessed. In particular, the POI influences the risk of residual disease after surgical tumor excision. OSCC POI ranges from solid “pushing border-like” infiltration to highly infiltrative growth, with spidery-like low-density tumor strands. Even small tumor islands/satellites isolated from the main tumor can sometimes be found at more than 1 mm from the main tumor, threatening the resection margins and requiring meticulous microscopical assessment, sometimes assisted with specific immunostainings (Figure S1). As such, the worst POI has been reported as an independent prognostic factor for disease-free survival in early stage OSCC^6^ and early stage oral tongue SCC.^7^ Consequently, in routine practices, surgeons aim for margins of 1 cm surrounding the assumed tumor, with the intention to obtain at least 5 mm of histopathological margin.

Electrocautery is a frequently used surgical modality for oral cavity resections, mainly due to its hemostatic function, critical to cauterize highly vascularized tissues of the oral cavity. This modality leads to the byproduction of electrosurgical vapors. Recently, the direct analysis of these vapors by rapid evaporative ionization mass spectrometry (REIMS) has shown great potential toward intraoperative recognition of tumor and healthy tissues.^8,9^ Molecules present in the vapors originated from tissue debris are ionized by REIMS, creating near real-time metabolic profiles. These REIMS metabolic profiles have been reported to be tissue-specific for different tumor and normal tissue types.^10−12^ Tissue specimens were cauterized *ex vivo* and analyzed by REIMS to establish libraries of tissue-specific metabolic profiles. The classifications obtained by REIMS metabolic profiles were then compared to their subsequent histopathologic examination. During histopathological examination, a trained pathologist deduces the evaporated tissue components by analyzing the histology surrounding the tissue defects that remain after cautery-sampling for REIMS analysis. These *ex vivo* libraries aim toward confident *in vivo* classification of normal and tumor tissues when the surgeon is resecting the tumor.^13,14^ The implementation of such technology adapted to routine surgical tools would provide direct feedback on tissue pathology without changing the surgical workflow. This provides unique, locally specific molecular information in support of the intraoperative decision-making process. Real-time recognition of tumor tissues could even lead to achieve finer, more precise, but still radical resection margins to improve the quality of life of patients with oral cavity cancers.

The ability of REIMS to detect small amounts of tumor cells (i.e., from islands of 15 tumor cells to single cell invasion, defining the two worst POI) is critical to assist oral cavity surgery.^6,15−17^ This is particularly important given the challenges associated with the OSCC POI. We propose to evaluate the sensitivity of REIMS, which has not been investigated so far. The versatility of REIMS has already been widely documented on diverse samples ranging from biological tissues^8,10,11^ to cell pellets.^18^ Contrary to complex and heterogeneous human cancerous tissues that require the estimation of the tissue components removed by electrocautery, cell lines constitute relatively homogeneous biological samples that allow the establishment of libraries of purer and well-controlled metabolic profiles. Therefore, a dedicated methodology for preparation of cell pellets assists with the assessment of the sensitivity of REIMS and its ability to detect a small amount of tumor cells in cell mixtures. This critical assessment is a next step toward safer oral cavity cancer resection with a more precise information source for intraoperative decision-making. In addition, we propose to study the heterogeneity of the metabolic profiles of tumor and normal oral cavity tissues composed of different tissue types (e.g., muscle, smaller structures like nerves), as these could affect the variance of the estimated tissue-specific metabolic profiles based on the histology surrounding the needle electrode-sampling defects for REIMS analysis.

Desorption electrospray ionization mass spectrometry (DESI-MS) is an ambient ionization source similar to REIMS and uses charged solvent droplets to desorb and ionize molecules from a surface.^19^ DESI-MS imaging (DESI-MSI) can generate two-dimensional spatially resolved metabolic distributions from frozen tissue sections. Combination of DESI-MSI analysis with subsequent histological staining of tissue sections allows extraction of tissue-specific metabolic profiles. DESI-MSI has been commonly employed on different pathological applications.^20−22^ As such, DESI-MSI on tongue cancer revealed greater intensity of cholesterol sulfate in normal mucosa compared to OSCC,^23^ previously hypothesized as biomarker for normal keratinocyte squamous maturation.^24^ Therefore, DESI-MSI complements REIMS as a molecular pathology tool to study oral cavity metabolic profiles and unravel the complex pathobiology of head and neck cancers, ranging from precursor lesions to field cancerization, and potentially improve surgical decisions.^25^

Here, we investigate the potential and identify the challenges of the application of REIMS analysis of electrosurgical vapors for oral cavity cancer surgery. First, we use REIMS analysis of electrosurgical vapors *ex vivo* to establish tissue-specific metabolic profiles with histopathology and validate our profiles with *in vivo*, real-time tissue analysis during patient surgeries. DESI-MSI is subsequently used to characterize the metabolic heterogeneity of resected oral cavity tissues. Finally, we assess the sensitivity of the REIMS technology by measuring metabolic profiles on cell pellets composed of mixtures of OSCC with normal keratinocytes or myoblasts.

## Materials and Methods

### Patients

This study included 11 patients who underwent surgery at Maastricht University Medical Center (MUMC+) between June 2017 and July 2019, and who gave written informed consent. Patients older than 18 years were eligible if they were planned for surgical resection of OSCC. Clinical and pathological data can be found in Supporting Information, Tables S1–S3. The study was approved by the Medical Ethics Committee of MUMC+ (approval number METC 16–4–168) and conducted according to the revised version of the Declaration of Helsinki.

### Tissue Preparation for *Ex Vivo* REIMS and DESI-MSI Analysis

The resection specimen was transferred fresh from the operating theater to the pathology department as soon as possible. A pathologist dissected normal and tumor tissue surplus to diagnostic needs from the resection specimen for the present study. Tissue slices were used for either immediate REIMS analysis or frozen in liquid nitrogen and stored at −80 °C for later experiments.

### *Ex Vivo* REIMS Tissue Analysis

Tissues were cauterized *ex vivo* using a monopolar handpiece (iKnife disposable device, Waters Research Center (WRC), Budapest, Hungary) equipped with a 1.7 mm diameter needle electrode, connected to an electrosurgical heat-generator (Force FX, Covidien), operated in cut pure mode. The generated vapors were aspirated though the REIMS interface via a venturi pump built-in into the mobile Xevo G2-XS Q-ToF mass analyzer (Waters Corporation, Wilmslow, U.K.). Isopropanol (Honeywell) containing leucine-encephalin (Sigma-Aldrich) was infused at 150 μL/min for lock mass correction.^26^ Acquisition parameters of REIMS data were: negative ionization mode, mass range: *m*/*z* 100–1500, scan time: 1 s. Mass resolution for [LeuEnk–H]^−^ was around 40000. After REIMS analysis, the remaining tissue was either fixed in formalin and embedded in paraffin, or frozen in liquid nitrogen. Hematoxylin and eosin (H&E) stained sections were prepared for histopathology review. The tissue surrounding the defect from the REIMS procedure was analyzed to deduce the tissue components of the evaporated tissue. The most substantial tissue component was taken as representative for the metabolic profile(s) generated from the tissue defect.

### DESI-MSI Tissue Section Analysis

Frozen tissues were sectioned using a cryotome (Microm) at 10 μm thickness, thaw mounted on histological glass slides (Superfrost), and stored at −80 °C prior to analysis. Experiments were performed on a DESI source equipped with a third generation prototype DESI sprayer, installed on a Xevo G2-XS Q-ToF MS (Waters Corporation, Wilmslow, UK). Methanol/water (98/2; Biosolve Chimie SARL) at 1.5 μL/min was used as a solvent. Transfer line was heated to 400–500 °C. Acquisition parameters were: negative ionization mode, mass range: *m*/*z* 100–1500, pixel size: 30 × 30 μm^2^, scan rate: 150 μm/sec. Mass resolution for [raffinose–H]^−^ was around 20000. After analysis, tissue sections were H&E stained and scanned on a slide scanner (Aperio CS2, Leica). Groups of six adjacent pixels were combined in histological areas selected by a pathologist (ImageScope v12.4.3.5008, Leica) to generate a DESI-MS metabolic profile (e.g., as nerve, tumor or muscle) (HD Imaging v1.5, Waters Corporation, Wilmslow, U.K.) to study the tissue heterogeneity.

### *In Vivo* REIMS Analysis

*In vivo* measurements were performed on a mobile Xevo G2-XS Q-ToF mass analyzer equipped with a REIMS source (Waters Corporation, Wilmslow, U.K.) in operating rooms at MUMC+ during four partial glossectomy procedures. Surgeries were performed using commercial hand-piece (Erbe) and heat generator (Valleylab FT10, Covidien). Electrosurgical vapors were directed toward the mass spectrometer with an identical source as for the *ex vivo* experiments. Acquisitions were performed as described for the *ex vivo* REIMS tissue analysis.

### Preparation of Cell Pellets and REIMS Cell Pellets Analysis

**Figure 1:**
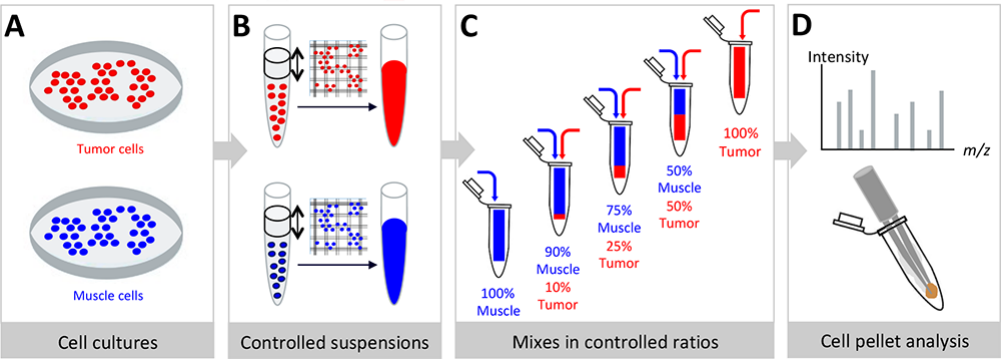
Preparation of cell pellets for REIMS analysis. (A) Different cell lines were cultured in separated conditions. (B) Based on the estimated cell concentration using a Bürker counting chamber, cell suspensions were diluted to produce cell suspensions with controlled cell concentrations. (C) Cell suspensions from different cell lines were mixed in controlled ratios (50/50, 75/25, 90/10). (D) After centrifugation and storage, cell pellets were analyzed by REIMS analysis of electrosurgical vapors to attribute metabolic profiles to the mixtures of cell lines.

Details related to the cell culture of human cell lines can be found in the Supporting Information. Cells at 70–90% confluency were washed with a phosphate-buffer solution (PBS) and detached using trypsin to create single cell suspension. Cells were counted using a Bürker counting chamber and diluted to obtain a suspension of 20 × 10^6^ cells per mL. This cell suspension was subsequently diluted in different ratios (i.e., 50/50, 75/25, 90/10) to obtain cell line mixes in 1.5 mL Eppendorf tubes, as illustrated in Figure 1. Cell line dilutions were well mixed and centrifuged to create a cell pellet. Supernatant medium was removed, and cell pellets were snap-frozen in liquid nitrogen and stored at −80 °C prior to analysis. Cell pellets were acclimated at room temperature for 2–5 min before REIMS analysis and cauterized using a cell line handpiece (WRC, Budapest, Hungary), connected to an electrosurgical heat-generator (Force FX, Covidien), operated in cut pure mode in an ML-1 laboratory. The generated vapors were aspirated into a benchtop REIMS Xevo G2-XS Q-ToF mass analyzer (Waters Corporation, Wilmslow, U.K.) in an API laboratory. Acquisitions were performed as described for the *ex vivo* REIMS tissue analysis.

### Data Analysis

REIMS and DESI-MS data were analyzed using a prototype of abstract model builder software (AMX v1.01563.0, WRC, Budapest, Hungary). Model preprocessing included 0.1 binning, lock-mass correction, background subtraction, and normalization. Unsupervised principal component analysis (PCA) was used for data reduction to obtain an overview of the variance of the metabolic profiles. Supervised principal component analysis-linear discriminant analysis (PCA-LDA) was used to optimize the separation between the profiles from different classes and to build classifiers (Supporting Information, Table S4). PCA-LDA and leave-one patient out cross-validation were used to compare the accuracy between MS-based classification and histopathological classification of biological tissues. For the REIMS *ex vivo* tissue analysis, one mass spectrum per sampling spot was selected for the tissue analysis over the mass range *m*/*z* 100–1500 with excluding leucine-encephalin associated peak mass ranges (*m*/*z* 554–558, *m*/*z* 594–597, *m*/*z* 1109–112, *m*/*z* 1131–1137) and was considered outlier if the mass spectrum deviated 5× standard deviation (SD). For the REIMS cell lines analysis: scans at the top of the sampling spot signal in the ion chromatogram were selected for the cell analysis over the mass range *m*/*z* 600–1000 and were considered outlier if they deviated 25× standard deviation (SD). PCA-LDA and leave-one biological replicate out cross-validation were used to compare the accuracy between MS-based classification and known prepared composition of cell pellet mixtures. Each passaged cell group was considered as a biological replicate. The sensitivity of the approach, defined as the minimum percentage of tumor cells detected in the dilution series, was based on receiver operating characteristic (ROC) curve analysis. The ROC curve was calculated based on predicted value. The iterative calculation compared the results of the prediction model as generated by the AMX software with the “clinical truth” (i.e., a “positive test” [value = 1] corresponded to the known presence of tumor cells in the dilution point). In order words, since the dilution series were generated with known compositions, the different mixture compositions were annotated as follow to generate the initial model for this calculation: (a) the “true” class was assigned to muscle only when no tumor cells were present is the mixture; and (b) “T” for tumor when at least 10% of tumor cells were introduced in the mixture. For the first iteration, the true positive for “muscle” cells corresponded to 0% of tumor cells, so 100% muscle cells only; therefore, any data point containing any tumor cells and predicted/classified as “muscle” were considered as false negative. For the second iteration, the threshold for “muscle” cells was increased to 10%, which means, all the data points including 10% of tumor cells that were predicted as “muscle” cells were considered as correctly classified. The corresponding file with the confusion matrix and an example of the calculation is provided in the Supporting Information, data file F1. The best cutoff was selected with the highest true positive rate together with the lowest false positive rate. An area under the curve (AUC) of 0.916 ± 0.015 was obtained using IBM SSPS statistical analysis software version 25 (IBM, Armonk, NY, U.S.A.). DESI-MSI data were converted to IMZML format in HD Imaging (v1.5, Waters Corporation, Wilmslow, U.K.) and imported into SCiLS Lab MVS (v2020a, SCiLS, Bruker, Bremen, Germany). The average spectra of the data set was exported to mMass 5.5.0^27^ for peak-picking including baseline correction and deisotoping. Image registration between DESI molecular images and corresponding histological images was performed for the selection of tissue-specific metabolic profiles to study tumor heterogeneity. Segmentation using K-means clustering was performed to obtain the specific metabolic profiles of nervous tissue.

## Results

### Establishment of a Tissue Classifier by *Ex Vivo* REIMS Analysis

**Figure 2:**
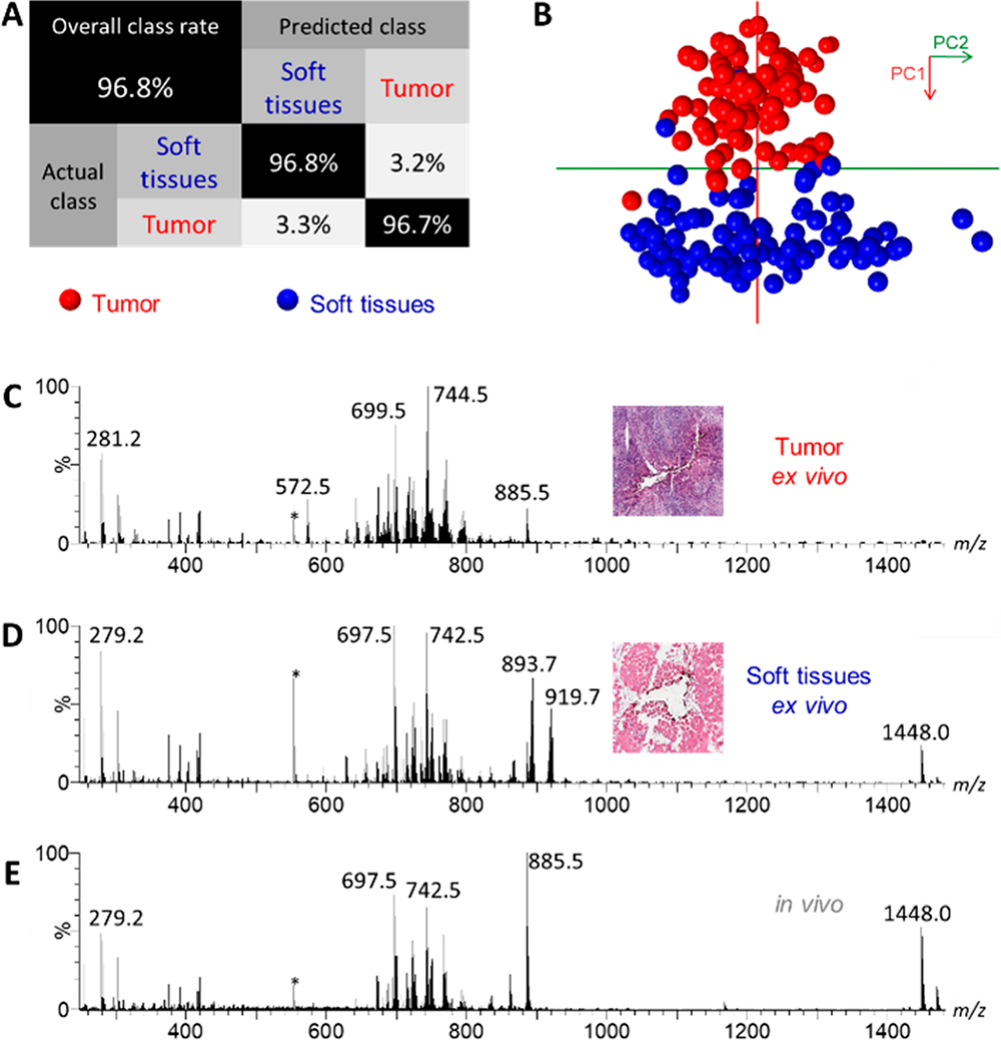
REIMS analysis of electrosurgical vapors of tongue tissues. 185 REIMS metabolic profiles (94 soft tissue and 91 tumor) were generated *ex vivo* on tissues provided by five patients and analyzed by REIMS on the mass range *m*/*z* 100–1500. Lock-mass leucine-encephalin is visible as *m*/*z* 554.3 (*). (A) Confusion matrix with predicted class by REIMS metabolic profiles and actual class defined by histopathology. (B) Principal component analysis score plot (PC1 which explains 73.7% of the variance of the data, PC2: 16.5%). (C) Representative REIMS metabolic profile for tumor tissue measured *ex vivo* generated in cut mode. (D) Representative REIMS metabolic profile for soft tissue measured *ex vivo* generated in cut mode. (E) REIMS metabolic profile measured *in vivo* generated in cut mode.

We used *ex vivo* REIMS analysis of electrosurgical vapors to build a library of 185 tissue-specific metabolic profiles on oral cavity tissues provided from 5 patients and reached a cross-validation accuracy of 96.8% (Figure 2A, Table S4, data matrix in file F2). The tissue sampling during REIMS analysis with a needle electrode led to the production of tissue defects. These defects presented themselves as circular holes of variable diameters (ca 1–1.5 mm) on the tissue sections intended for pathologist examination. Tumor and soft tissue profiles were clearly separated along the PC1 axis in the PCA score plot (Figure 2B). This allowed to target molecular identification strategies for mass features associated with these tissue types using PC1 mass feature loading plot (Figure S2 and Table S6). Representative metabolic profiles for soft tissue (e.g., muscle, adipose) and tumors show distinctive profiles (Figure 2C,D) and are similar to *in vivo* metabolic profiles (Figure 2E). REIMS analysis of electrosurgical vapors measured *in vivo* led to the generation of intense ion signals compatible with direct tissue recognition (Figure S3).

### Assessment of Metabolic “Hot-Spots” in Oral Cavity Tissues by DESI-MSI Analysis

**Figure 3:**
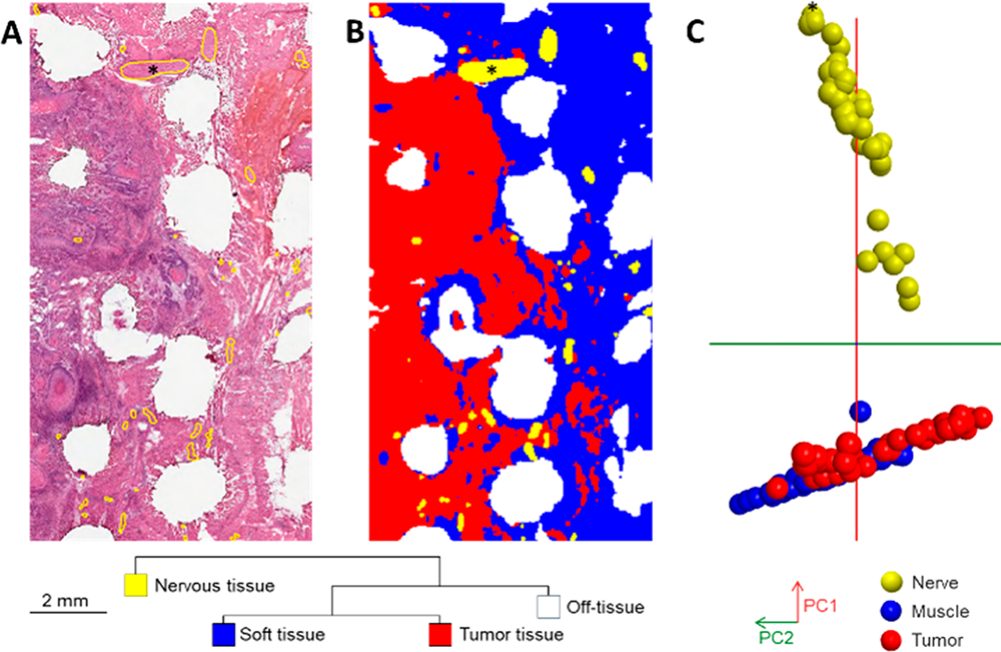
Distinct nerve metabolic profiles in oral cavity tissues by DESI-MSI. DESI-MS profile of nerve area indicated by an asterisk (*) indicated in Supporting Information, Figure S4B and position on PCA score plot in Figure 2C. (A) Histology surrounding tissue defects of needle electrode-sampling for REIMS analysis surrounded by nerve features delineated in yellow on one resected specimen. (B) Segmentation analysis discriminating nervous tissue from the rest of the imaged areas based on DESI-MS profiles. (C) Principal component analysis score plot of DESI-MS profiles (55 nerve, 54 muscle, and 53 tumor) from tissue provided by six patients on the mass range *m*/*z* 600–1000 (PC1, which explains 80.3% of the variance of the data; PC2, 7.8%).

Next, we used DESI-MSI to investigate if histological features, potentially missed from the needle electrode-sampling methodology, could affect the variance of metabolic profiles. Segmentation analysis (Figure 3A,B), PCA (Figure 3C) and relative confusion matrix (Figure S4C) showed that nerve metabolic profiles are clearly distinctive from muscular and tumor metabolic profiles. Figure S4B,C and Table S6 showed tentative identification of mass features characteristic of DESI-MS metabolic profiles associated with nerve tissue.

### Investigation of Tumor Metabolic Markers in Oral Cavity Tissues by DESI-MSI Analysis

**Figure 4:**
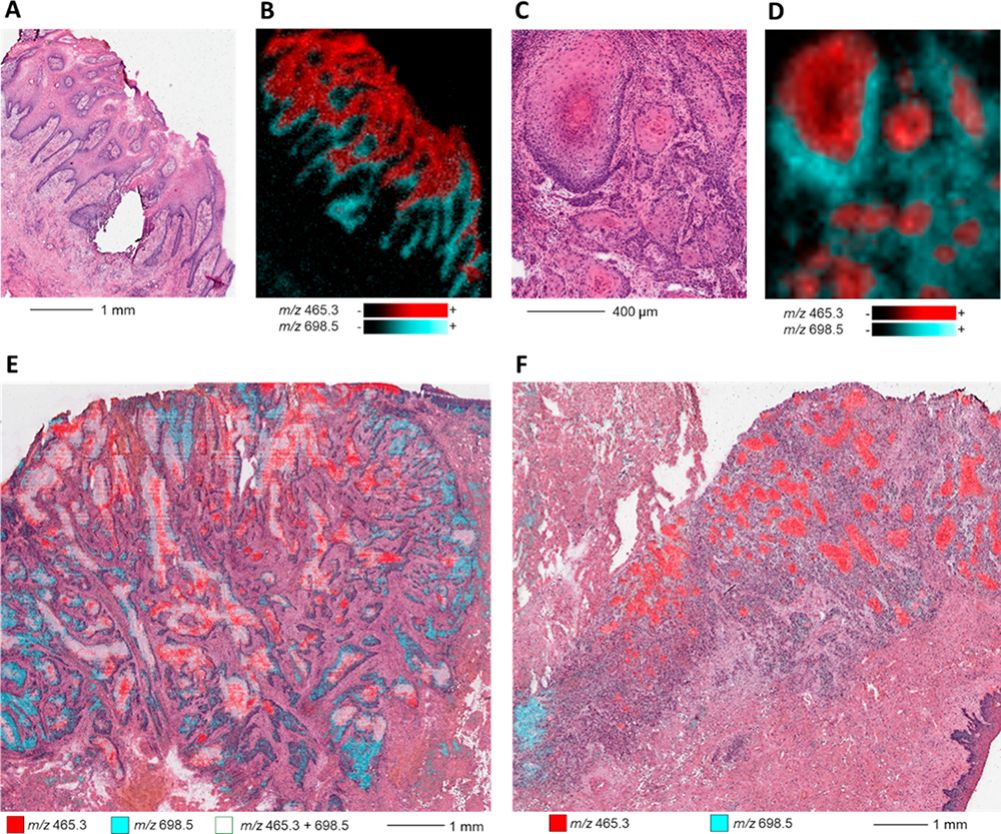
Common metabolic markers for mucosa and oral squamous cell carcinoma (OSCC) differentiation by DESI-MSI acquired with 30 × 30 μm^2^ pixel size. Extracted ion images display distribution for ether-phosphatidylethanolamine PE(O-16:1/18:2) (indicated with *m*/*z* 698.5) and cholesterol sulfate (at *m*/*z* 465.3). (A) H&E staining of a physiological hyperplastic dorsal tongue mucosa. (B) Overlaid distributions of ether-phosphatidylethanolamine PE(O-16:1/18:2), mainly localized in the basal basaloid part, and cholesterol sulfate, mainly in the apical spinous part of the dorsal tongue mucosa. (C) H&E staining in an oral squamous cell carcinoma (OSCC) with gradual differentiation from basaloid (basophilic stained cells) part to spinous (eosinophilic stained cells) part. (D) Overlaid distributions of cholesterol sulfate mainly in the basaloid part and ether-phosphatidylethanolamine PE(O-16:1/18:2) mainly in the spinous part of this moderately differentiated OSCC. (E) Predominant distribution of cholesterol sulfate in the central keratinizing part (abrupt formation of keratin pearls) and predominant distribution of ether-phosphatidylethanolamine PE(O-16:1/18:2) in the proliferative border part of a basaloid-type OSCC. (F) Predominant distribution of cholesterol sulfate in the keratinizing part and in keratin pearls and predominant distribution of ether-phosphatidylethanolamine PE(O-16:1/18:2) in the proliferative border part of a spinous OSCC.

Tumor heterogeneity is a consistent concern in cancer diagnostics. Here, we searched for markers reflective of OSCC metabolic heterogeneity using DESI-MSI. OSCC can present different degrees of differentiation along with different degrees of keratinization. Typically, well-differentiated OSCC closely mimics the maturation of squamous epithelia as seen in oral cavity mucosa. Therefore, we explored the metabolic changes associated not only to squamous maturation (in a hyperplastic dorsal normal oral mucosa), but also to OSCC squamous differentiation and keratinization (in an OSCC with gradual differentiation). We screened mass features discriminative of basaloid/spinous (i.e., basal/apical in normal mucosa) changes using PCA and tissue-specific molecular distributions (Figures S5–S7). The most obvious intramucosal metabolic shift was from ether-phosphatidylethanolamine PE(O-16:1/18:2) (at *m*/*z* 698.5) localized at the basal side to cholesterol sulfate (at *m*/*z* 465.3) mainly localized at the apical side (Figure 4A,B, Figures S5, S6, S8–S10, and Table S6 for identification). This metabolic shift (*m*/*z* 698.5 to *m*/*z* 465.3) was also observed in OSCC displaying central keratinization features (Figure 4C,D, Figures S5 and S7)) from basaloid tumor into spinous tumor. Correlating cholesterol sulfate and ether-phosphatidylethanolamine PE(O-16:1/18:2) with histology showed the heterogeneous distributions of these metabolites in the non-keratinizing and keratinizing parts of basaloid OSCC (Figure 4E). Similar analysis showed only the distribution of *m*/*z* 698.5 in the central part of a spinous OSCC (Figure 4F). In addition, while cholesterol sulfate was intense in central scaring and keratinizing tumor parts, ether-phosphatidylethanolamine PE(O-16:1/18:2) was intense on the infiltrative proliferative tumor borders (Figure 4E,F).

More details on the observed metabolic shift can be found in Supporting Information, Figures S8–S10. However, these two metabolites detected by DESI-MSI did not contribute strongly to the REIMS metabolic profiles and did not influence the variance of the REIMS tumor profiles limiting the extrapolation of our results to understand the heterogeneity of REIMS metabolic profiles. Comparison of REIMS and DESI-MS metabolic profiles was performed using tissue classifications predicted by REIMS and DESI-MS metabolic profiles on each other (Figure S11, Supporting Information, file F3).

### Assessment of REIMS Sensitivity by Measuring Metabolic Profiles in Cell Pellets

**Figure 5:**
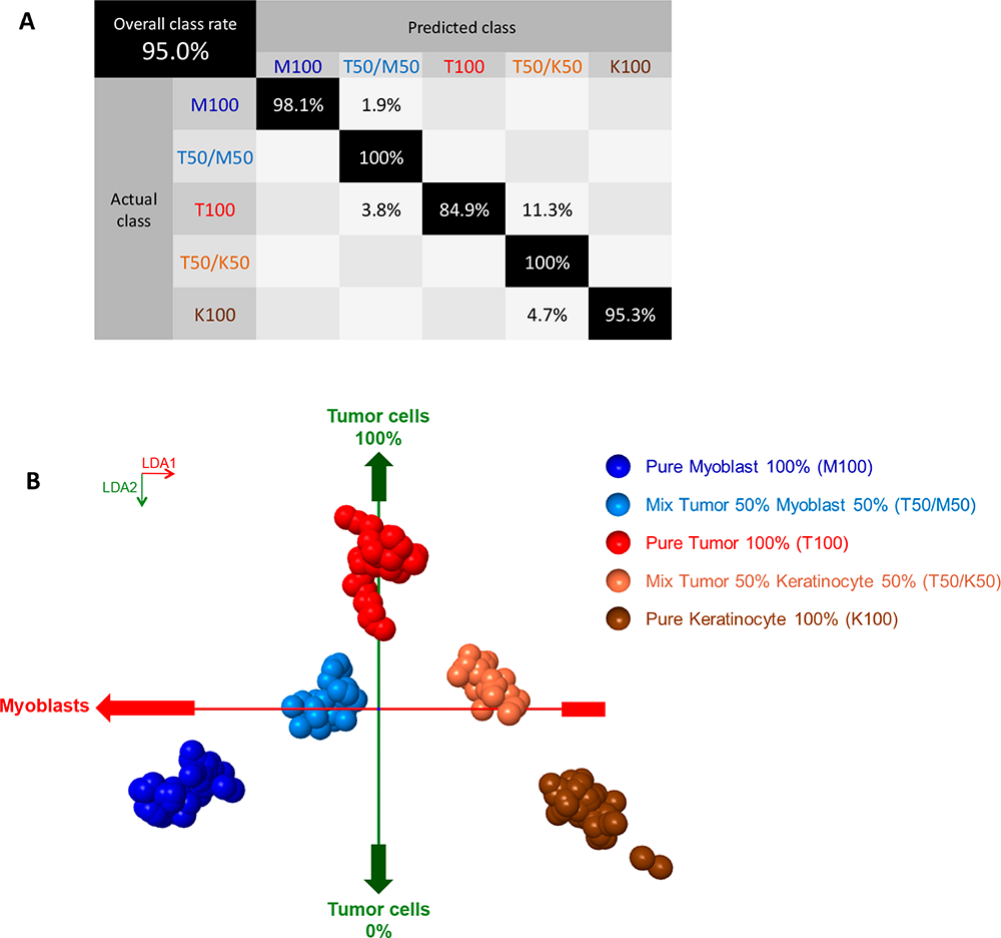
Evaluation of the REIMS sensitivity by measuring electrosurgical vapors generated from cell pellets. (A) Confusion matrix with predicted class by REIMS metabolic profiles and actual class defined by cell pellet preparation, based on 220 REIMS metabolic profiles (53 myoblast 100%, 44 tumor/myoblast 50/50, 53 tumor 100%, 27 tumor/keratinocyte 50/50, 43 keratinocyte 100%) were generated from three biological replicates for each class (except only two biological replicates for tumor/keratinocyte 50/50; mass range *m*/*z* 600–900, 10 PC, 4 LDA). (B) Pseudo–LDA score plot relative to (A).

The possible presence of tumor satellites in the resection margins of OSCC challenges the sensitivity of REIMS technology. To assess this sensitivity, we used mixtures of cell lines to represent the oral cavity tissue environment; that is, keratinocytes for the mucosa and myoblasts for the muscle, and OSCC cell lines for the tumor. A total of 220 REIMS metabolic profiles (53 myoblast 100%, 44 tumor/myoblast 50%/50%, 53 tumor 100%, 27 tumor/keratinocyte 50%/50%, 43 keratinocyte 100%) were measured from three biological replicates for each class (except only 2 biological replicates for tumor/keratinocyte 50%/50%). These metabolic profiles were properly recognized reaching a cross-validation of 95% accuracy (Figure 5A). Representative metabolic profiles for each class display some progressive changes between classes (Figures S12–S14 and Figure 5B).

**Figure 6:**
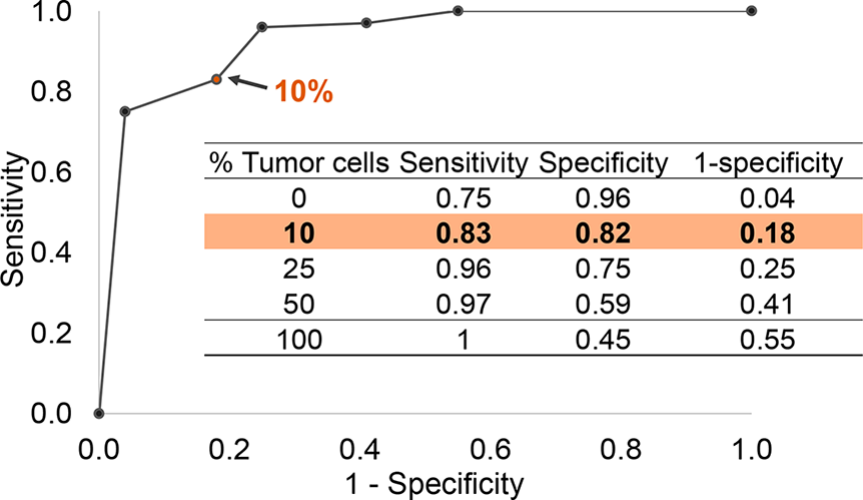
ROC curve for the evaluation of the REIMS sensitivity by measuring electrosurgical vapors generated from dilutions of cell pellets tumor/myoblasts.

A total of 336 REIMS metabolic profiles (93 tumor 100%, 93 myoblast 100% from 5 replicates each, and 44 tumor 50%/myoblast 50%, 57 tumor 25%/myoblast 75%, 49 tumor 10%/muscle 90% from 3 replicates each) were used to build a model with two classes: tumor (i.e., tumor from 100% to 10% were assigned to “tumor”) and muscle (i.e., muscle 100% only). These metabolic profiles were classified with a correct classification rate of 79,8% accuracy. Representative metabolic profiles, a PCA score plot, and cross-validation data matrix can be found in Figures S9 and S11 and Supporting Information, data file F1, respectively. We used the results obtained from this classification results to build a ROC curve for more detailed evaluation of the sensitivity (Figure 6, Supporting Information, data file F1). We determined that our REIMS approach was capable to detect tumor cells at a proportion of 10% when mixed myoblasts, with a 83%-true positive rate. Complementary analysis of the exact composition of the mixed cell pellets with linear combination of REIMS pure metabolic profiles, and with cytospin analysis by MSI are available in Supporting Information (Table S7 and Figure S15).

## Discussion

Our tissue classifier based on *ex vivo* REIMS metabolic profiles enabled the recognition of tumor and soft tissues with an overall 96.8% accuracy. This demonstrates the potential of REIMS to provide rapid, *in situ* assessment of tissues during oral cavity surgeries. We estimated that REIMS can detect tumor cells down 10% of the burned area with 83% sensitivity (true positive rate) and 82% specificity. This offers perspectives for tumor cell detection in highly infiltrative malignancies such as OSCC. This approach is expected to support surgical decision-making and improve precise determination of resection margins to spare healthy and functional tissue. This would greatly benefit patients and result in a better quality of life and potentially prevent unnecessary additional oncological treatment. The way toward successful clinical implementation of real-time metabolic classifiers for radical oral cavity cancer resection must address several challenges that we would like to critically discuss in the next sections.

### Histological Attribution of Metabolic Profiles

In the present study, a needle electrode was employed to cauterize the tissues to generate libraries of tissue-specific metabolic profiles. This needle electrode is used in current clinical settings, hence the choice for this sampling tool. Consequently, a “hole”, caused by burning the tissue is created. This poses challenges for the pathologist to evaluate the content of the evaporated tissue, absent from the tissue section. So far, this assessment is solely based on an estimation of the tissue components surrounding the “holes” left by the sampling and deducing a description of the absent cells. In addition, in the current setting, the acquisition of metabolic profiles is uncorrelated to the exact volume or quantity of tissue cauterized during the sampling. Consequently, no precise metabolic profile can be attributed to a specific tissue class in a quantitative manner. For this reason, spatially resolved metabolic profiling using mass spectrometry imaging (e.g., DESI-MSI) provides additional and complementary information to REIMS and histopathology alone, starting by linking metabolic profiles with a tissue unit represented by a space unit, the pixel. Here, DESI-MSI suggests that small and scattered tissue features such as nerves can influence the metabolic profiles attributed to normal and tumor tissues. Nevertheless, the amount of nerve tissue present in the burnt holes is difficult to estimate due to their limited occurrence in the tissue and due to the inexistence of a pure REIMS metabolic profile specific to nerve tissue. Clinical MSI is now reaching cellular resolution, becoming an asset for molecular pathology workflows.^28^ As such, MSI is expected to play a critical role to support the establishment of sensitive real-time metabolic classifiers, with its advantages, especially the precise attribution of metabolic profiles for OSCC tumor islands in resection margins. More globally, MSI may also participate to better characterize OSCC pathology, with the detection not only of OSCC tumor islands but also of premalignant cells that can cause local relapse and secondary malignancies, which may lead to even more efficient resections.

### Assessment of the Sensitivity of REIMS for Biological Classification

Sensitivity of real-time metabolic classifiers has not been addressed so far. However, this is a critical aspect to consider when one does not want to leave tumor cells in a patient. As such, quantifying tumor cells detected in electrosurgical vapors during cauterization becomes a crucial criterion for implementing direct sampling technologies into clinical practice.^29^ This is especially true for challenging OSCC oral cavity cancer surgery where tissue sparing is paramount and the need for surgical precision is extremely high. Here, we addressed the sensitivity question for real-time tissue recognition for the first time. The classification of quantitative mixtures of different cells selected to mimic the tumor and the tissue composition of the human oral cavity (i.e., keratinocytes for mucosa, muscles for soft tissues) was used to associate values for sensitivity and specificity. Despite limitations (i.e., number of biological replicates, low similarity of metabolic profiles between biological tissues and cell lines), our cell assay constitutes a first step toward an assessment of the sensitivity of real-time metabolic classifiers. Classically employed to assess the performance (i.e., sensitivity, specificity) of diagnostic tests, the ROC curve was also employed in metabolomics to predict the ability to classify two disease states based on multiple biomarkers.^30,31^ The realization of a ROC curve from the cross-validation data of the recognition of cell mixtures further illustrates this consideration for sensitivity. Nevertheless, the conflicting qualitative versus quantitative association between a biological sample and metabolic profile persists. More precisely, there has been no assessment of the number of cells or the mass of biological tissue required to realize quantitatively metabolic profiling. Despite the indication of percentages of tissue recognition, these are uniquely based on representative pattern recognition associated with modern algorithms. Analytical platforms have exceled to provide quantitative and sensitive assessments for numerous and diverse biomedical applications by using standardized performance indicators such as the calibration line, and the limit of detection (i.e., the sensitivity in analytical chemistry, here referred to as the lowest percentage of tumor cells in mixture that can be detected). Mass spectrometry, as a modality that generates quantifiable signals, should be able to reach better quantitative and sensitive standards, even in real-time. Performance indicators should emerge to valorize intraoperative analyses into reliable sensitive diagnostic tests.

### Toward Targeted Metabolic Profiling for Sensitive Tissue Recognition

Many approaches for tissue recognition are based on untargeted metabolic profiles to screen tissue pathologies,^32^ even if a single metabolite can be specific for tumor tissues (e.g., cholesterol sulfate in prostate cancer, 2-hydroxybutyrate isocitrate dehydrogenase 1 mutant gliomas).^22,33^ Identification of potential specific metabolic markers representative of biological processes, as we reported with DESI-MSI for the characterization of OSCC keratinization, enables targeted approaches for metabolic profiling based on specific markers to reach higher sensitivity. Analysis of targeted metabolic profiling may therefore switch to mass spectrometers specially designed for utmost quantitative assessments for specific molecules and results in more sensitive tissue classifications.^34^ Tissue classification was already shown translational among different mass analyzers (Orbitrap, QTOF, ion-trap).^35^ In addition, these models of mass spectrometers (e.g., triple quadruple, ion trap) would be more suitable as portable for more realistic implementation in the operating room to support decision-making.

## Conclusion

We demonstrate that REIMS metabolic profiles, collected *ex vivo*, have huge potential to predict the histopathology of oral cavity cancer during surgical resection with high accuracy. We determined the sensitivity of REIMS technology using a novel approach based on assessment of cellular mixtures. Complementary spatially resolved MSI visualized metabolic and tumor-specific markers linked to tumor heterogeneity. Future research related to intraoperative diagnostics will benefit from these innovative quantitative strategies to evaluate real-time mass spectrometry-based metabolic profiling technologies sensitivity, especially for detection of OSCC cells in resection margins. The approach where REIMS and MSI data are compared, demonstrated to be a prerequisite to evaluate tissue heterogeneity, identify, and target specific metabolic tumor markers for improved tumor detection during surgery, which will ultimately benefit many patients undergoing surgical procedures.

## Supporting Information Available

Additional experimental details, materials, methods and supplementary results, including information on patient population characteristics, model metric parameters, molecular identification, confusion matrices, and discriminative mass features (PDF)
Data matrix generated via AMX; Sensitivity–ROC curve (XLSX)
