# Supplementary material for: Evaluation of the Sensitivity of Metabolic Profiling by Rapid Evaporative Ionization Mass Spectrometry: Toward More Radical Oral Cavity Cancer Resections
Source: Anal Chem. 2022 May 3;94(19):6939–47. doi: 10.1021/acs.analchem.1c03583 (PMC9118195; doi:10.1021/acs.analchem.1c03583)
Supplement: Supplementary file 1 — ac1c03583_si_001.pdf [file ac1c03583_si_001.pdf]

Supplementary material

Evaluation of the Sensitivity of Metabolic Profiling by Rapid Evaporative Ionization Mass Spectrometry: towards more Radical Oral Cavity Cancer Resections

Pierre-Maxence Vaysse[1,2,3], Imke Demers[2,4,5]‡, Mari F.C.M. van den Hout[4,5]‡, Wouter van de Worp[6], Ian G.M. Anthony[1], Laura W.J. Baijens[2,5], Bing I. Tan[2,5], Martin Lacko[2,5], Lauretta A. A. Vaassen[7], Auke van Mierlo[7], Ramon C. J. Langen[6], Ernst-Jan M. Speel[4,5], Ron M.A. Heeren[1]*, Tiffany Porta Siegel[1]*, Bernd Kremer[2,5]

1 Maastricht MultiModal Molecular Imaging Institute (M4i), Division of Imaging Mass Spectrometry, Maastricht University, Universiteitssingel 50, 6229 ER Maastricht, The Netherlands.

2 Department of Otorhinolaryngology, Head and Neck Surgery, Maastricht University Medical Center+, 6202 AZ Maastricht, The Netherlands.

3 Department of Surgery, Maastricht University Medical Center+, 6229 ER Maastricht, The Netherlands.

4 Department of Pathology, Maastricht University Medical Center+, 6202 AZ Maastricht, The Netherlands.

5 GROW School for Oncology and Developmental Biology, Maastricht University Medical Center+, 6202 AZ Maastricht, The Netherlands.

6 Department of Respiratory Medicine, NUTRIM School for Nutrition, Toxicology and Metabolism, Maastricht University Medical Center+, 6202 AZ Maastricht, The Netherlands.

7 Department of Cranio-Maxillofacial Surgery, Head & Neck Surgery, Maastricht University Medical Center+, 6202 AZ Maastricht, The Netherlands.

*Corresponding Authors: Tiffany Porta Siegel, M4i Institute, Division of Imaging Mass Spectrometry, Maastricht University, Universiteitssingel 50, 6229 ER Maastricht, The Netherlands; email: tiffany.porta@gmail.com; Ron Heeren, M4i Institute, Division of Imaging Mass Spectrometry, Maastricht University, Universiteitssingel 50, 6229 ER Maastricht, The Netherlands; email: r.heeren@maastrichtuniversity.nl.

Table of contents

Materials and Methods **3**
Cell culture of human cell lines. 3
Preparation of cell cytospin for MALDI-MSI analysis. 3
MALDI-MSI cell analysis. 3
Data analysis – details for the ROC curve analysis and explanation for supplementary file F1. 3
Molecular identification. 3
Tissue section immunostainings. 4
Linear combination of pure REIMS metabolic profiles to approximate the composition of mixed cell pellets. 4
Linear combinations of two pure REIMS metabolic profiles (e.g. cell pellet 100% tumor and cell pellet 100% myoblast) were employed to approximate the composition of the mixed cell pellets (e.g. cell pellet of 50% tumor – 50% myoblast) and therefore to characterize the data quality. 4
Annotations of basaloid tissue. 4

Supplementary Tables **5**
Supplementary Table S1. Demographic and pathological tumor characteristics of the patient population. 5
Supplementary Table S2. Study dedicated pathology assessment performed for patients included in REIMS ex vivo and DESI-MSI. 5
Supplementary Table S3. Utilization of the tissues of the patient population for mass spectrometric analyses. 5
Supplementary Table S4. Metric parameters of PCA and PCA-LDA models. Metrics are Euclidean in the PCA space. Mahalanobis distance is the metric in the LDA space. 6
Supplementary Table S5. Sampling scheme: number of metabolic profiles per patient and per tissue class for REIMS ex vivo analysis. 6
Supplementary Table S6. Molecular identification by REIMS and DESI-MS. The present list of molecular identifications is tentative based on mass accuracy after lock-mass correction and tandem mass spectrometry experiments in the absence of chromatographic separation.”. 7

Supplementary Table S7. Approximation of the composition of the mixed cell pellets based on linear combination of 2 pure REIMS metabolic profiles (for the dataset used in figure 5). 8

Supplementary Figures **9**

Supplementary figure S1. Histological features of oral squamous cell carcinoma of the tongue by immunostaining for cytokeratin 5-6 (A-B, from one patient) and P16 (C, from another patient). Positive areas are brown and indicate the localization of tumor cells. Inked resection margins are on the inked border on the right of sections on A and B. (A) Infiltrative pattern of invasion with spidery-like growth extensions (indicated by arrows) corresponding to the worst pattern of invasion (WPOI) type 4 defined as small tumor islands of 15 tumor cells or fewer per island at the tumor edge. (B) Presence of small tumor islands/microsatellites less than 1 mm from the resection margin (indicated by arrows; inked right border of the section) corresponding to WPOI type 5, which is defined by tumor satellites at 1 mm or more from the main tumor or next closest satellite. (C) Solid growth pattern with low-density tumor strands (<15 cells) at the infiltrative margin on the right (WPOI type 4). More details about the patterns of invasion can be found in references (5-8). 9

Supplementary Figure S2. PC1 mass features loading plot of the REIMS metabolic profiles ex vivo of tissues of figure 1. 10

Supplementary Figure S3. REIMS metabolic profiles of electrosurgical vapors analyzed in vivo during surgeries of patient 9 in coagulate mode (A) and of patients 10 (B) and 11 (C) in cut mode. Metabolic profiles includes *m/z* 893.7, 919.9 and 1448.0 mass peaks characteristic of soft tissues in our REIMS tissue classifier of tumor and soft tissues *ex vivo*. Lock-mass leucine-encephalin is at *m/z* 554.3. 10

Supplementary Figure S4. Analysis of nervous tissue by DESI-MSI (supplementary to fig. 3). 11

Supplementary Figure S5. Screening for mass features discriminative for basaloid/spinous changes, part I: principal component analysis on mucosa maturation and oral squamous cell carcinoma differentiation (supplementary to fig. 4). 12

Supplementary Figure S6. Screening for mass features discriminative for basaloid/spinous changes, part II: mucosa-specificity of molecular distributions (supplementary to fig. 4). H&E staining of a physicological hyperplastic dorsal tongue mucosa, with basaloid mucosal tissue annotated in yellow by the pathologist, and associated DESI-MSI molecular distributions for the screened discriminative features for basaloid/spinous changes. 14

Supplementary Figure S7. Screening for mass features discriminative for basaloid/spinous changes, part III: tumor-specificity of molecular distributions (supplementary to fig. 4). H&E staining of a squamous cell carcinoma with gradual differentiation, with basaloid tumor tissue annotated in yellow by the pathologist, and associated DESI-MSI molecular distributions for the screened discriminative features for basaloid/spinous changes. 15

Supplementary Figure S8. Fragmentation pattern of *m/z* 658.5 by DESI-MS/MS experiment. 16

Supplementary Figure S9. Intensity box plots of mass features m/z 465.3 (A) and m/z 698.5 (B) in normal mucosa (basaloid and spinous) and in oral squamous cell carcinoma viable tumor parts (basaloid, spinous, keratin) analyzed by DESI-MSI and normalized on total ion count. 18

Supplementary Figure S10. Intensity box plots of m/z 465.3 (A) and m/z 698.5 (B) in spinous oral squamous cell carcinoma (OSCC) viable tumor areas analyzed by DESI-MSI, normalized on total ion count for each patient. 18

Supplementary Figure S11. Classifier of REIMS and DESI-MS metabolic profiles. 19

Supplementary Figure S12. REIMS metabolic profiles of cell line mixes. 20

Supplementary Figure S13. PCA score plot related to Fig. 5. 21

Supplementary Figure S14. (A) PCA score plot related to the model used to calculate the ROC curve. (B) Same score plot but using a different color coding to localize the data with 100% (red), 50% (pink), 25% (yellow), 10% (green) tumor cells and 100% muscle cells. 21

Supplementary Figure S15. Cytospin slide preparation to assess the quality of the cell line mixtures. Example of a mixture tumor/myoblast cells shows clumping of one type of cells on the left bottom corner potentially indicating an partially unhomogenous cell pellet preparation. 22

References for the supplementary material **23**

Materials and Methods

Cell culture of human cell lines.

The human tongue squamous cell carcinoma cell line UT-SCC-5 (RRID:CVCL_7858) was cultured in Dulbecco's modified Eagle's medium (DMEM, Thermo Fisher Scientific ; #31966021) supplemented with 10% (v/v) fetal bovine serum (FBS) and 1x non-essential ammino acids (Thermo Fisher Scientific; # 11140050).The normal oral keratinocyte (NOK) cell line (kindly provided by Karl Munger, Tufts University Medical School, Boston, MA, USA), was cultured in keratinocyte serum-free medium (KSFM) supplemented with 2.6 µg/mL bovine pituitary extract (BPE) and 0.16 ng/mL recombinant epidermal growth factor (rEGF) (Thermo Fisher Scientific ; #17005075).

Human immortalized C25 myoblasts [1] were kindly provided by K. Mamchaoui and V. Mouly (Platforme MyoLine, Institute of Myology, Paris). Cells were cultured in Dulbecco's modified Eagle's medium (DMEM) 4.5 g/L glucose, GlutaMAX™ (Gibco; #61965-026) supplemented with 9% (v/v) fetal bovine serum (FBS), 50 U/mL penicillin and 50 µg/mL streptomycin (P/S) (Thermo Fisher Scientific; #15140-122), 10 µg/ml insulin (Sigma; #91077C), 1 ng/mL human basic fibroblast growth factor (Milipore Merck; #GF003AF), 10 ng/mL epidermal growth factor (Sigma; #E5036), 0.5 µg/mL dexamethasone (Sigma; #D4902) and 50 µg/mL fetuin (Sigma; #F2379). All cell lines were cultured at 37 °C in a humidified atmosphere with 5% CO_2.

Preparation of cell cytospin for MALDI-MSI analysis.

Cells were mounted on indium tin oxide (ITO)-coated glass slides (Delta Technologies, USA) using a Cyto-Tek cytocentrifuge (Miles scientific), model no. 4332. The slides were then stored at -80 until utilization for MALDI-MSI. ITO glass slides were successively washed for a few minutes in a xylene bath, then in ethanol bath and dried in a desiccator before use. Five µl of a mixed cell suspension was diluted in 190 µl 4% paraformaldehyde (PFA) and fixated for 15 minutes at room temperature to allow their utilization outside of a ML-II laboratory.

MALDI-MSI cell analysis.

Sample was taken out of a low-temperature freezer and dried for a couple of minutes in a desiccator. 50 mg of crystalline norharmane (Sigma-Aldrich, The Netherlands) dissolved in MS grade methanol (Biosolve Chimie SARL, France) was used as a matrix to dry coat the sample using a Sublimator (HTX Technologies, USA). Sublimation was set at 140 degrees for 180 seconds. Experiments were performed on a rapifleX MALDI-ToF (Bruker Daltonik GmbH, Germany). Measurements were performed in negative ionization mode, over the mass range *m/z* 600 to 1000, set at 5 x 5 µm pixel size, at 100 shots per position.

Data analysis – details for the ROC curve analysis and explanation for supplementary file F1.

The dilution series were generated with known composition, the different mixture compositions were annotated as follow to generate the initial model for this calculation: a) the "true" class was assigned to muscle only when no tumor cells were present is the mixture (i.e. marked as "MUSC100" for 100% muscle cells); and b) "T" for tumor when at least 10% of tumor cells were introduced in the mixture (column D). For the first iteration, the true positive for "muscle" cells corresponded to 0% of tumor cells, so 100% muscle cells only (Column G); therefore, any metabolic profile containing any tumor cells and predicted/classified as "muscle" was considered as false negative (results in table starting in column K9). For the second iteration, the threshold for "muscle" cells was increased to 10%, which means, all the metabolic profiles including 10% of tumor cells that were predicted as "muscle" cells were considered as correctly classified (see results in the table starting in column K347).

Molecular identification.

REIMS and DESI-MS spectra were lock-mass corrected on deprotonated leucine-encephalin *m/z* 554.2609 $[M-H]^-$ and deprotonated raffinose *m/z* 503.1606 $[M-H]^-$ respectively for identification based on mass accuracy. Tandem mass spectrometry experiments were performed by collision-induced dissociation with argon gas. Experimental data were tested on ALEX^{123} lipid calculator [2] for lipids, and compared to reference literature [3].

Tissue section immunostainings.

Tissue section immunostainings for cytokeratins and P16 from patients included in the study were kindly provided by the department of pathology (MUMC+) and scanned on a slide scanner (Aperio CS2, Leica, USA).

Linear combination of pure REIMS metabolic profiles to approximate the composition of mixed cell pellets.

Linear combinations of two pure REIMS metabolic profiles (e.g. cell pellet 100% tumor and cell pellet 100% myoblast) were employed to approximate the composition of the mixed cell pellets (e.g. cell pellet of 50% tumor – 50% myoblast) and therefore to characterize the data quality.

The intensities of each mass feature of each pure REIMS metabolic profile (i.e. 100% tumor, 100% keratinocyte, 100% muscle) were averaged to generate a representative pure REIMS metabolic profile for each category (i.e. 3 in total, 100% tumor, 100% keratinocyte, 100% myoblast). Normalization was performed by dividing the intensity of each mass feature of the metabolic profile by the intensity of the highest intensity mass feature within the metabolic profile. Linear equations were established between 2 representative pure REIMS metabolic profiles (i.e. between 100% tumor and 100% keratinocyte, between 100% tumor and 100% myoblast).

Each mixed REIMS metabolic profile was approximated using two normalized "pure" metabolic profiles multiplied by coefficients. For example, the 50% tumor 50% myoblast metabolic profile was approximated by metabolic profiles from 100% tumor and 100% myoblast. The two coefficients (one coefficient for the 100% tumor metabolic profile and one coefficient for the 100% myoblast metabolic profile) were then transformed into percentages by simple summation and division (i.e. % tumor = 100% * tumor coefficient / (tumor coefficient + myoblast coefficient)).

Annotations of basaloid tissue.

The H&E images of supplementary figures S6 and S7 were annotated for basaloid tissue by a pathologist on QuPath (v.0.2.0.m8).

Supplementary Tables

Supplementary Table S1. Demographic and pathological tumor characteristics of the patient population.

Patient	Sex	Age	Site	Tumor	Infiltrative pattern
1	Female	76	Oral cavity, cheek	Squamous cell carcinoma	Yes
2	Male	59	Oropharynx, tonsil	Squamous cell carcinoma	Yes
3	Male	63	Oral cavity, anterior floor of the mouth	Squamous cell carcinoma	No
4	Female	44	Oral cavity, lateral tongue	Squamous cell carcinoma	Yes
5	Male	80	Oral cavity, lateral tongue	Squamous cell carcinoma	No
6	Male	64	Oral cavity, mobile tongue	Squamous cell carcinoma	Yes
7	Male	75	Oral cavity, base of tongue	Squamous cell carcinoma	Yes
8	Female	44	Oral cavity, lateral tongue	Squamous cell carcinoma	Yes
9	Female	74	Oral cavity, lateral tongue	Squamous cell carcinoma	Yes
10	Male	62	Oral cavity, lateral tongue	Squamous cell carcinoma	Yes
11	Male	63	Oral cavity, lateral tongue	Squamous cell carcinoma	Yes

Supplementary Table S2. Study dedicated pathology assessment performed for patients included in REIMS ex vivo and DESI-MSI.

Patient	Differentiation	Keratinization
1	Spinous	+
2	Spinous	+
3	Spinous	+++
4	Basaloid and spinous	+
5	Spinous	-
6	Spinous	+
7	Basaloid	-
11	Basaloid	+

Supplementary Table S3. Utilization of the tissues of the patient population for mass spectrometric analyses.

Patient	REIMS *in vivo*	REIMS *ex vivo*	DESI-MSI
1			X
2			X
3		X	
4		X	X
5		X	X
6		X	X
7		X	
8	X		
9	X		
10	X		
11	X		X

Supplementary Table S4. Metric parameters of PCA and PCA-LDA models. Metrics are Euclidean in the PCA space. Mahalanobis distance is the metric in the LDA space.

Classifier	Tumor vs. Soft tissues	Nerve vs. Tumor vs. Muscle	Basaloid vs. Spinous	Tumor vs. Soft tissues	Tumor vs. Keratinocyte vs. Myoblast	Tumor vs. Myoblast
Number of classes	2	3	3	2	5	5
Ionization	REIMS	DESI-MS	DESI-MS	REIMS & DESI-MS	REIMS	REIMS
Sample	Tissues	Tissues	Tissues	Tissues	Cells	Cells
Model	PCA-LDA	PCA-LDA	PCA	PCA-LDA	PCA-LDA	PCA-LDA
Number of PC	15	3	3	15	10	10
Number of LDA	1	2	-	1	4	1
Cross-validation	Yes	Yes	No	Yes	Yes	Yes
Variance of PC1	27.79%	80.34%	59.84%	49.03%	52.98%	35.32%
Variance of PC2	17.09%	7.80%	18.64%	12.83%	14.92%	31.17%
Variance of PC3	13.92%	2.92%	6.01%	7.58%	12.28%	12.56%
Variance of PC4	9.89%	-	-	5.88%	5.39%	7.08%
Variance of PC5	6.76%	-	-	3.71%	4.06%	4.18%
Variance of PC6	4.58%	-	-	3.39%	2.51%	2.46%
Variance of PC7	3.41%	-	-	2.20%	1.90%	1.90%
Variance of PC8	2.55%	-	-	1.77%	1.19%	0.99%
Variance of PC9	2.24%	-	-	1.62%	0.71%	0.75%
Variance of PC10	1.42%	-	-	1.28%	0.53%	0.62%
Variance of PC11	1.13%	-	-	0.98%	-	-
Variance of PC12	0.95%	-	-	0.81%	-	-
Variance of PC13	0.68%	-	-	0.62%	-	-
Variance of PC14	0.66%	-	-	0.56%	-	-
Variance of PC15	0.62%	-	-	0.48%	-	-

Supplementary Table S5. Sampling scheme: number of metabolic profiles per patient and per tissue class for REIMS ex vivo analysis.

Patient	Tumor tissues	Soft tissues	Total
P3	7	6	13
P4	17	14	31
P5	9	21	30
P6	15	42	57
P7	46	8	54
Total	94	91	220

Supplementary Table S6. Molecular identification by REIMS and DESI-MS. The present list of molecular identifications is tentative based on mass accuracy after lock-mass correction and tandem mass spectrometry experiments in the absence of chromatographic separation.”.

Ion source	Class	Identification	Adduct	Formula	Tissue	PCA *m/z*	Measured *m/z*	Theoretical *m/z*	Mass error (ppm)	Related fragment ions upon MS/MS
REIMS	Fatty acid (FA)	Palmitic acid - FA 16:0	$[M-H]^-$	$C_{16}H_{31}O$	Soft tissue	255.2	255.2319	255.2330	4.1	-
REIMS	Fatty acid (FA)	Linoleic acid - FA 18:2	$[M-H]^-$	$C_{18}H_{31}O_2$	Soft tissue	279.2	279.2333	279.2330	1.2	-
REIMS	Fatty acid (FA)	Oleic acid - FA 18:1	$[M-H]^-$	$C_{18}H_{33}O_2$	Soft tissue	281.2	281.2476	281.2486	3.6	-
REIMS	Fatty acid (FA)	Arachidonic acid - FA 20:4	$[M-H]^-$	$C_{20}H_{31}O_2$	Soft tissue	303.2	303.2337	303.2330	2.5	-
REIMS	Ceramide (Cer)	Cer 34:1	$[M+Cl]^-$	$C_{34}H_{67}NO_3{}^{[35]}Cl$	Tumour	572.4	572.4802	572.4815	2.3	536.5
REIMS	Ceramide phosphate (CerP)	CerP 36:2;2	$[M-H]^-$	$C_{36}H_{69}NO_6P$	Tumour	642.4	642.4866	642.4868	0.3	79.0
REIMS	Phosphatidyl ethanolamine (PE)	PE 32:0	$[M-NH_3-H]^-$	$C_{37}H_{70}O_8P$	Tumour	673.4	673.4815	673.4814	0.2	255.3
REIMS	Phosphatidyl ethanolamine (PE)	PE 34:3	$[M-NH_3-H]^-$	$C_{39}H_{69}O_{10}$	Tumour	697.4	697.4802	697.4814	1.7	661.4, 435.2, 279.3, 255.3
REIMS	Phosphatidic acid (PA) / Phosphatidyl ethanolamine (PE)	PA 36:2 / PE 34:1	$[M-H]^-$ / $[M-NH_3-H]^-$	$C_{39}H_{72}O_8P$	Tumour	699.4	699.4971	699.4970	0.1	281.3, 255.3
REIMS	Phosphatidyl ethanolamine (PE) / Phosphatidyl choline (PC)	PE 36:2 / PC 34:2	$[M-H]^-$ / $[M-CH_3-H]^-$	$C_{41}H_{77}NO_8P$	Soft tissue	742.5	742.5364	742.5392	3.8	742.5, 706.5, 480.3, 279.3, 255.3
REIMS	Phosphatidyl ethanolamine (PE)	PE 36:1 / PC 34:1	$[M-H]^-$ / $[M-CH_3-H]^-$	$C_{41}H_{79}NO_8P$	Tumour	744.5	744.5519	744.5549	4.0	708.4, 480.3, 281.3, 255.3
REIMS	Triacylglycerol (TAG)	TAG 52:2	$[M+Cl]^-$	$C_{55}H_{102}O_6{}^{[35]}Cl$	Soft tissue	893.7	893.7311	893.7370	6.6	857.6, 281.3
REIMS	Triacylglycerol (TAG)	TAG 54:3	$[M+Cl]^-$	$C_{57}H_{104}O_6{}^{[35]}Cl$	Soft tissue	919.7	919.7469	919.7527	6.3	883.5, 303.3
REIMS	Cardiolipin (CL)	CL 72:8	$[M-H]^-$	$C_{81}H_{141}O_{17}P_2$	Soft tissue	1447.9	1447.9677	1447.9650	1.9	695.5, 415.3, 279.3
DESI-MS	Ether phosphatidyl ethanolamine (PE)	PE O-34:2	$[M-H]^-$	$C_{39}H_{75}NO_7P$	Nerve	700.5	700.5263	700.5287	3.4	436.3, 418.3, 281.3
DESI-MS	Ether phosphatidyl ethanolamine (PE)	PE O-36:2	$[M-H]^-$	$C_{41}H_{79}NO_7P$	Nerve	728.5	728.5568	728.5600	4.3	281.3
DESI-MS	Cholesterol	Cholesterol sulfate	$[M-H]^-$	$C_{27}H_{45}O_4S$	Spinous	-	465.3019	465.3044	5.4	96.6
DESI-MS	Ether Phosphatidyl ethanolamine (PE O-)	PE O-16:1/18:2	$[M-H]^-$	$C_{39}H_{73}NO_7P$	Basaloid	-	698.5098	698.5130	4.6	436.3, 418.3, 279.2

Supplementary Table S7. Approximation of the composition of the mixed cell pellets based on linear combination of 2 pure REIMS metabolic profiles (for the dataset used in figure 5).

Biological cell pellet mixes	Theoretical expected mean (expressed in % of tumor profile)	Empirical mean ± standard deviation (expressed in % of tumor profile)
Mix 50% tumor – 50% keratinocyte	50%	46% ± 8%
Mix 50% tumor – 50% myoblast	50%	19% ± 5%

The mean and the standard deviation of these experimental data are shown on table S7. The metabolic profiles of the mixes 50% tumor – 50% keratinocyte were more proportional to the theoretical composition of their pure metabolic profiles (46% ± 8% for 50% of tumor profile) than the metabolic profiles of the mixes 50% tumor – 50% myoblast (19% ± 5% for 50% of tumor profile) of their respective pure metabolic profiles. These results led to hypothesize that when mixed at 50%, tumor cells and keratinocytes tend to generate metabolic profiles of similar intensities, while myoblasts tend to generate less intense metabolic profiles than tumor cells

Supplementary Figures

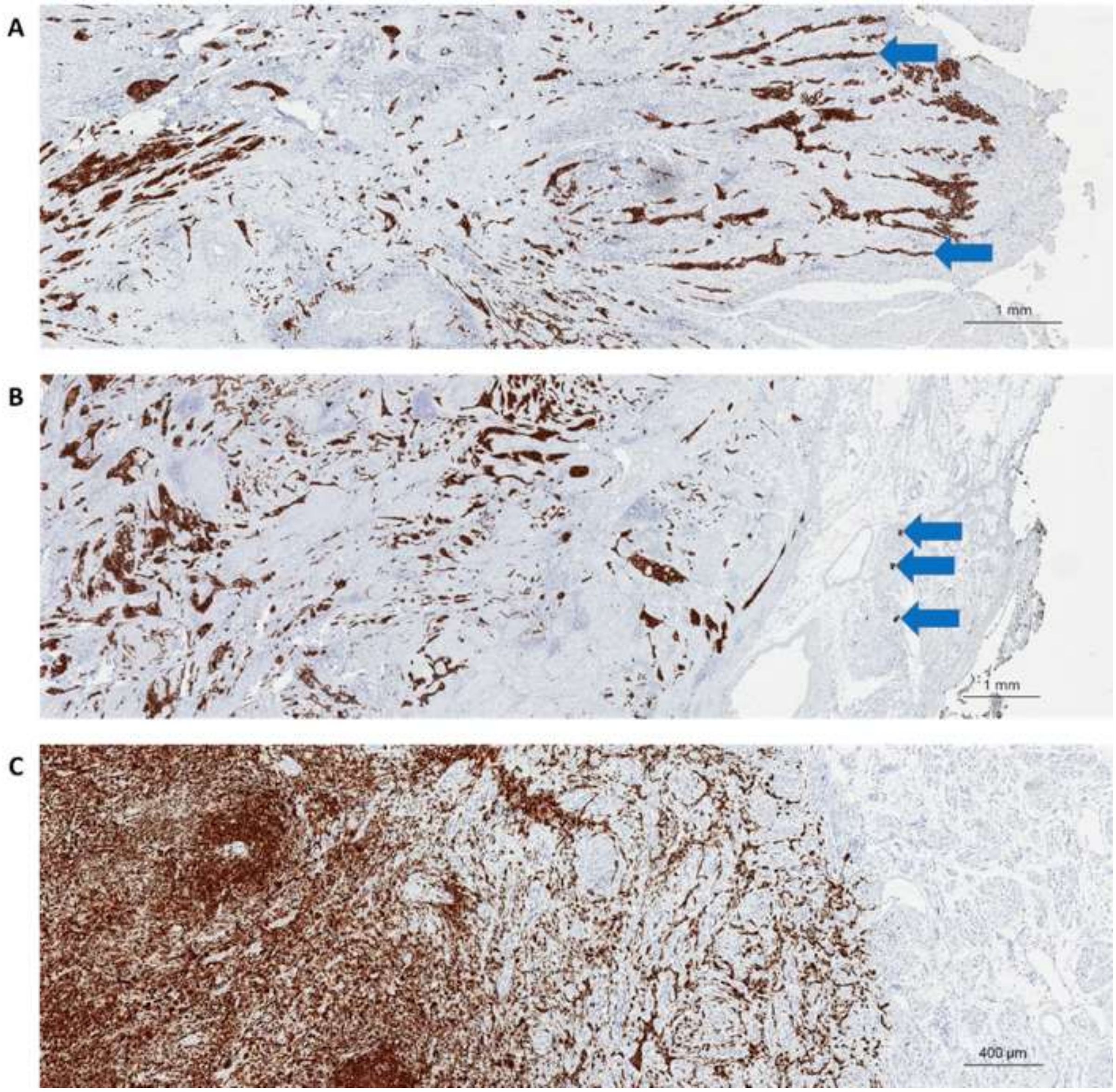

Supplementary figure S1. Histological features of oral squamous cell carcinoma of the tongue by immunostaining for cytokeratin 5-6 (A-B, from one patient) and P16 (C, from another patient). Positive areas are brown and indicate the localization of tumor cells. Inked resection margins are on the inked border on the right of sections on A and B. (A) Infiltrative pattern of invasion with spidery-like growth extensions (indicated by arrows) corresponding to the worst pattern of invasion (WPOI) type 4 defined as small tumor islands of 15 tumor cells or fewer per island at the tumor edge. (B) Presence of small tumor islands/microsatellites less than 1 mm from the resection margin (indicated by arrows; inked right border of the section) corresponding to WPOI type 5, which is defined by tumor satellites at 1 mm or more from the main tumor or next closest satellite. (C) Solid growth pattern with low-density tumor strands (<15 cells) at the infiltrative margin on the right (WPOI type 4). More details about the patterns of invasion can be found in references [4].

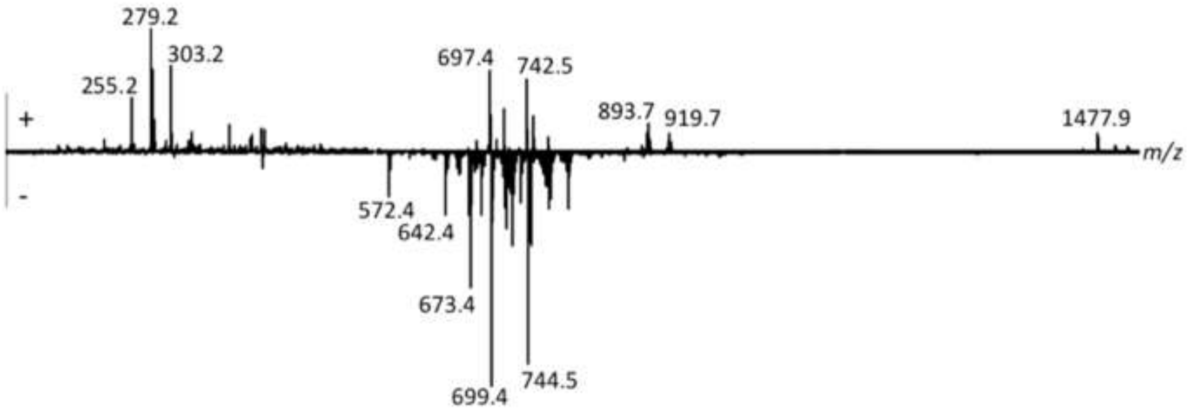

Supplementary Figure S2. PC1 mass features loading plot of the REIMS metabolic profiles ex vivo of tissues of figure 1.

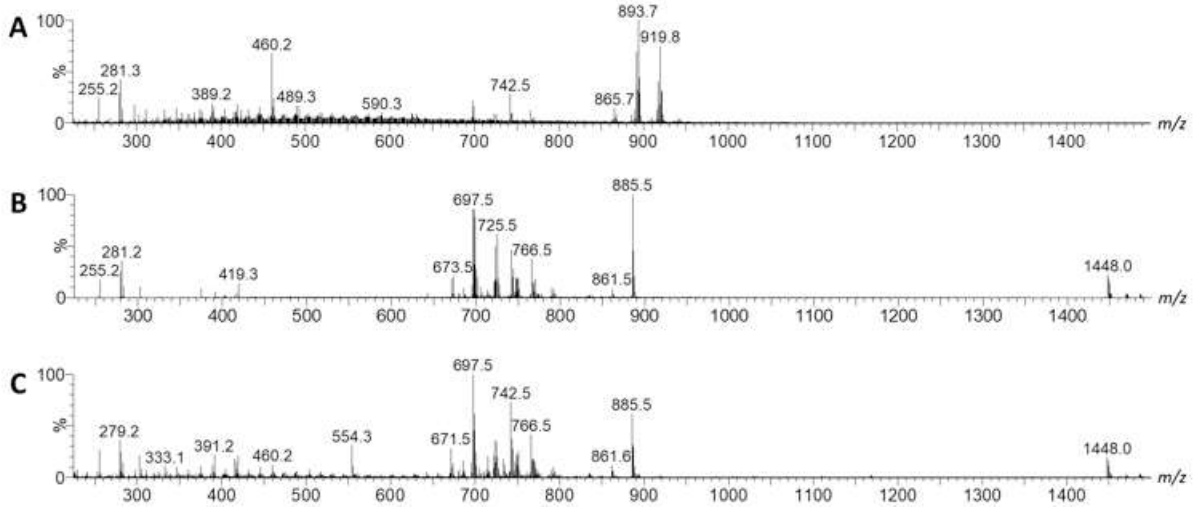

Supplementary Figure S3. REIMS metabolic profiles of electrosurgical vapors analyzed in vivo during surgeries of patient 9 in coagulate mode (A) and of patients 10 (B) and 11 (C) in cut mode. Metabolic profiles includes *m/z* 893.7, 919.9 and 1448.0 mass peaks characteristic of soft tissues in our REIMS tissue classifier of tumor and soft tissues *ex vivo*. Lock-mass leucine-encephalin is at *m/z* 554.3.

A

Overall class rate		Predicted Class		
86.8%		Muscle	Nerve	Tumor
Actual class	Muscle	94.5%	0%	5.6%
	Nerve	0%	94.5%	0%
	Tumor	34.0%	0%	66.0%

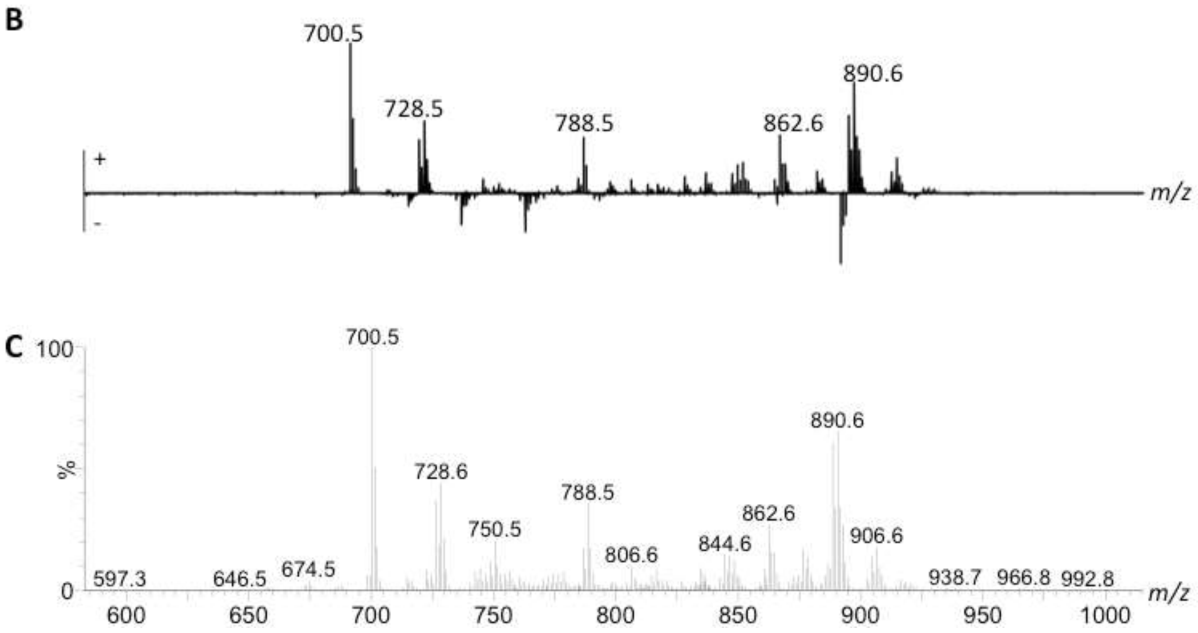

Supplementary Figure S4. Analysis of nervous tissue by DESI-MSI (supplementary to fig. 3).

(A) Confusion matrix for the classification of muscle, nerve and tumor tissue with predicted class by DESI-MS metabolic profiles and actual class defined by histopathology.

(B) PC1 mass features loading plot.

(C) Representative DESI-MS metabolic profile of nervous tissue. Selected from area indicated by an asterisk on fig. 2.

Nerve tissue profiles are fully properly recognized by the DESI-MS classifier (except for 2 outlayers), while the classification of tumor and muscle tissue is less accurate.

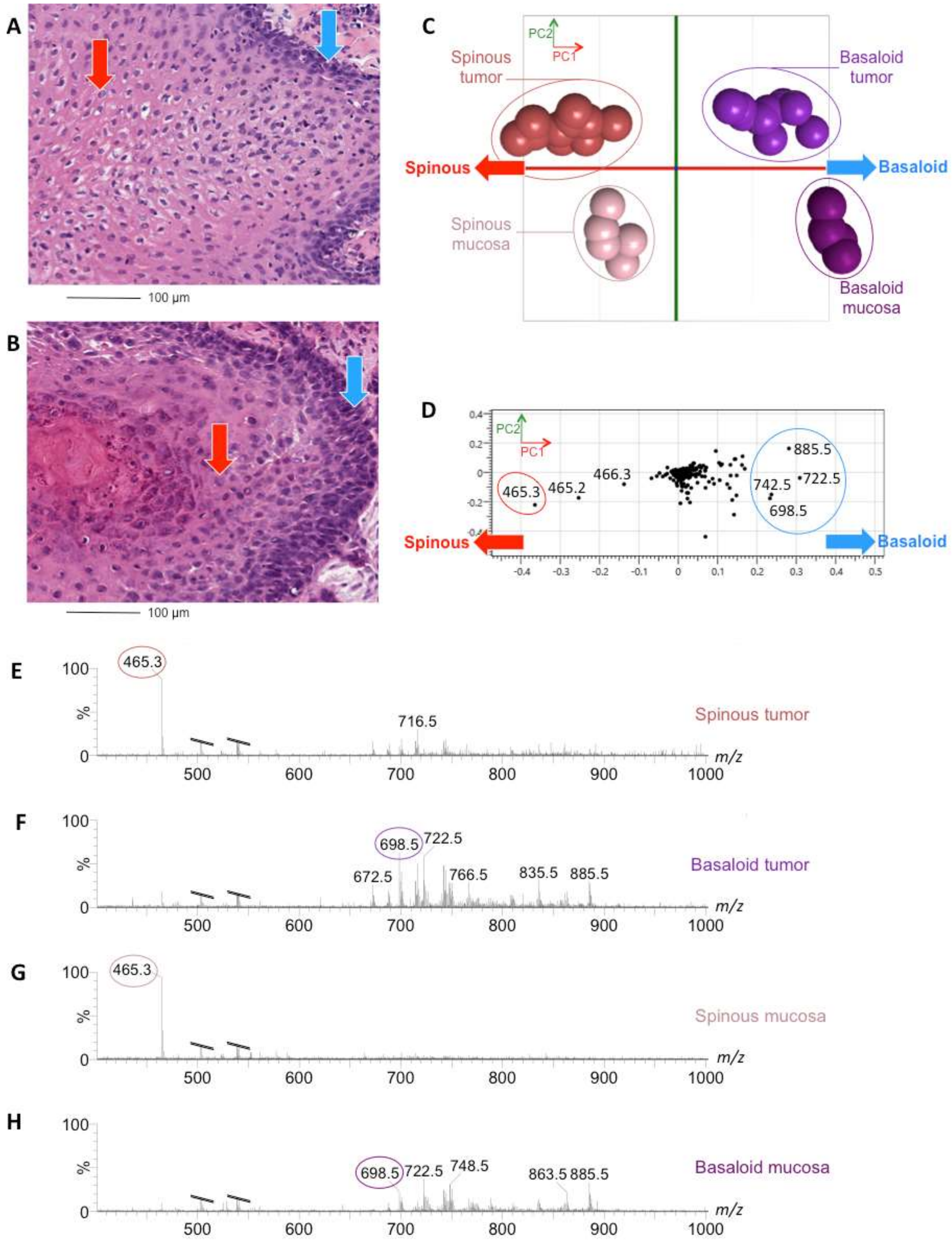

Supplementary Figure S5. Screening for mass features discriminative for basaloid/spinous changes, part I: principal component analysis on mucosa maturation and oral squamous cell carcinoma differentiation (supplementary to fig. 4).

(A-B) Histological changes from basaloid cells (basophilic, indicated by blue arrows) to spinous (eosinophilic, indicated by red arrows) cells.

- (A) Histological changes during physiological mucosa maturation.
- (B) Histological changes during oral squamous cell carcinoma differentation.

(C-D) Screening for basaloid/spinous mass features of interest by DESI analysis of tongue tissues.

(C) PCA score plot of 34 DESI-MS profiles (10 basaloid mucosa, 10 spinous mucosa, 5 basaloid tumor, 9 spinous tumor) from tissue provided by 2 patients on the mass range *m/z* 400-1000 (PC1 which explains 59.9% of the variance, PC2 18.6%).

(D) PC1/PC2 mass features loading plot of PCA in (C).

(E-H) Representative DESI-MS metabolic profile for spinous tumor (E), basaloid tumor (F), spinous mucosa (G) and basaloid mucosa (H). Mass peaks attributed to lock-mass raffinose were removed.

While mass feature 465.3 appears as the main discriminative mass feature for spinous tissue, 4 mass features – 698.5, 722.5, 742.5, 885.5 – appeared as discriminative for basaloid tissue (Fig. **S5D**).

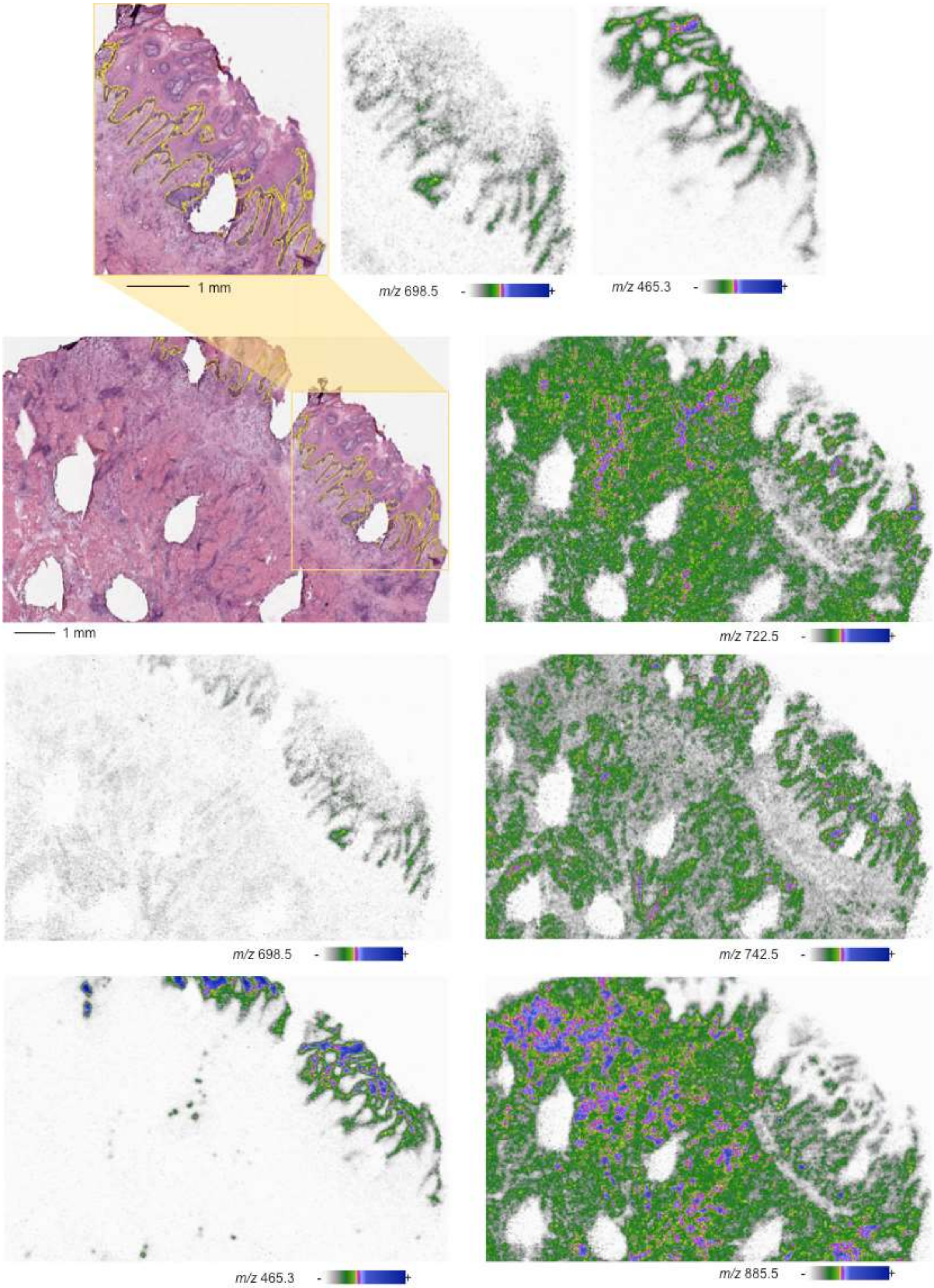

Supplementary Figure S6. Screening for mass features discriminative for basaloid/spinous changes, part II: mucosa-specificity of molecular distributions (supplementary to fig. 4). H&E staining of a physicological

hyperplastic dorsal tongue mucosa, with basaloid mucosal tissue annotated in yellow by the pathologist, and associated DESI-MSI molecular distributions for the screened discriminative features for basaloid/spinous changes.

Among the mass features discriminative for basaloid/spinous changes highlighted by PCA (Fig. **S5D**), molecular distributions of *m/z* 722.5, *m/z* 742.5 and *m/z* 885.5 appeared to be non specific for basaloid mucosal tissue. However, the features at *m/z* 698.5 and *m/z* 465.3 appeared specific for basaloid and spinous mucosal tissue respectively and were therefore considered the most suitable mass features to discriminate basaloid/spinous changes in normal mucosa.

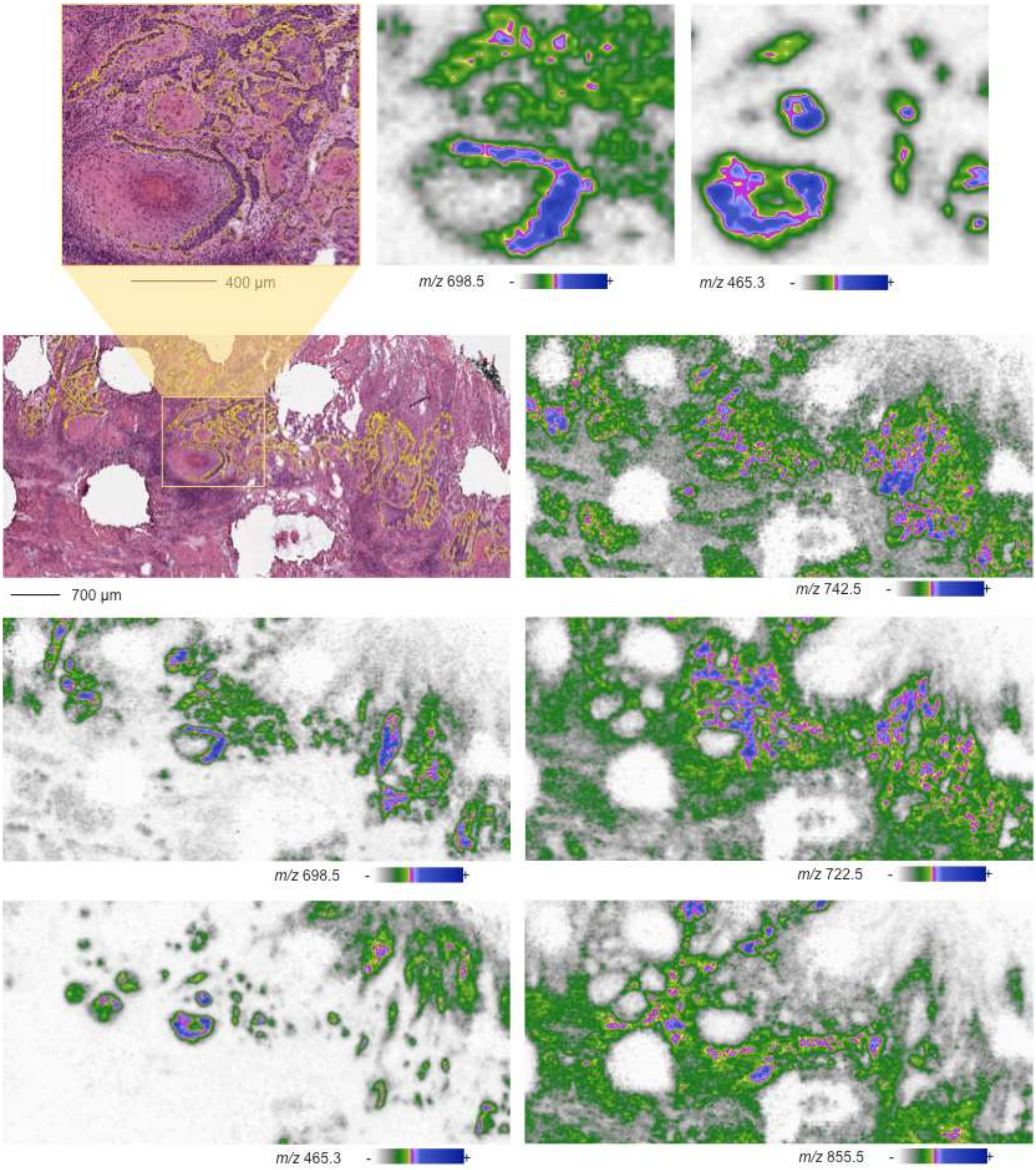

Supplementary Figure S7. Screening for mass features discriminative for basaloid/spinous changes, part III: tumor-specificity of molecular distributions (supplementary to fig. 4). H&E staining of a squamous cell carcinoma with gradual differentiation, with basaloid tumor tissue annotated in yellow by the pathologist, and associated DESI-MSI molecular distributions for the screened discriminative features for basaloid/spinous changes.

Among the mass features discriminative for basaloid/spinous changes highlighted by PCA (Fig. **S5D**), molecular distributions of *m/z* 722.5, *m/z* 742.5 and *m/z* 885.5 appeared non specific for basaloid tumor. However, *m/z* 698.5 and *m/z* 465.3 appeared specific for basaloid and spinous mucosal tissue respectively and were therefore considered the most suitable mass features to discriminate basaloid/spinous changes in OSCC.

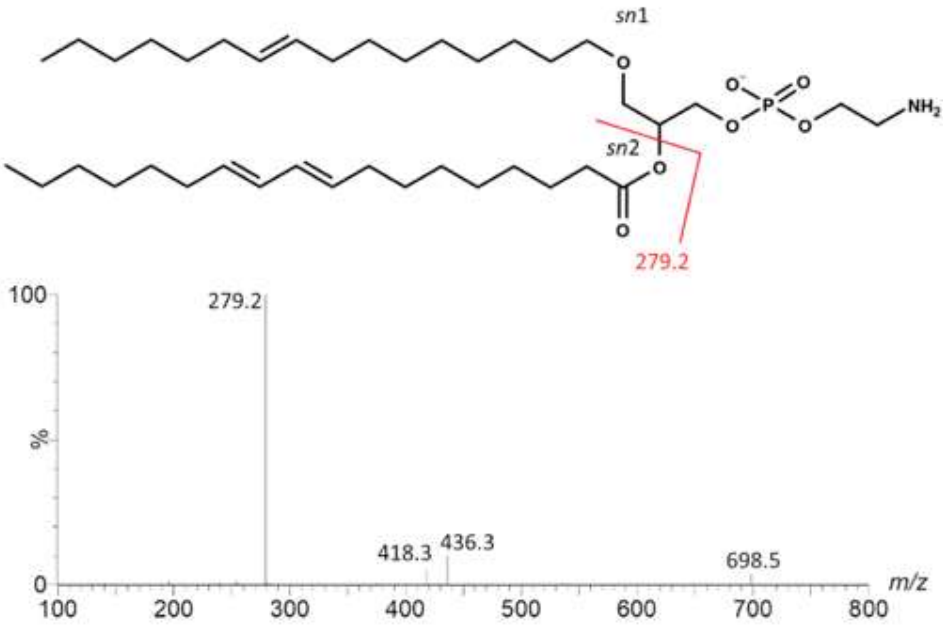

m/z 279.2	*sn2* RCOO⁻ ion
m/z 418.2	Neutral loss of *sn*2 RCOOH group from [M-H]⁻
m/z 436.3	Loss of *sn*2 acyl chain as ketene RCH=C=O from [M-H]⁻
m/z 698.5	Precursor ion[M-H]⁻

Supplementary Figure S8. Fragmentation pattern of *m/z* 658.5 by DESI-MS/MS experiment.

Search for tumor metabolic markers in oral cavity tissues by DESI-MSI analysis – details

SCC can show different forms of differentiation (from basaloid to spinous) defined by the morphological characteristics of the tumor cells. Basaloid differentiation is characterized by the resemblance of tumor cells to basal cells in normal squamous epithelium (i.e. relative high nuclear/cytoplasm ratio). Spinous differentiation is characterized by the resemblance of tumor cells to the cells of the spinous layer of normal squamous epithelium (i.e. cohesive cells with cell to cell bridges and ample, deeply eosinophilic cytoplasm). Keratinisation which is the progressive formation of keratin (deeply eosinophilic acellular material, end-product of the squamous differentiation) in the tumor can be part of tumor differentiation.

Comparison of precisely extracted metabolic profiles of central viable tumor showed the similar trend of metabolic shift in normal mucosa and OSCC based on the intensity changes of *m/z* 465.3 and *m/z* 698.5 with differentiation/keratinization (Supplementary Fig. **S9A-B**). Comparison of OSCC-metabolic profiles suggested that the metabolic shift is more in line with the keratinization status than with the squamous differentiation as one of the spinous OSCC presented a very low keratinization status and had the particularity to have a very low intensity level of cholesterol sulfate (Supplementary Fig. **S10A-B**). Nevertheless, extrapolation of these results should be limited regarding our small sized-dataset. In that context, worth mentioning that while the progressive keratinization appeared with a decrease of cholesterol sulfate in the keratin center (Fig. **4C-D**), abrupt keratinization in keratin pearls formation was still characterized by intense cholesterol sulfate (Fig. **4E-F**), which may indicate a slightly different metabolic process between these two histopathological entities.

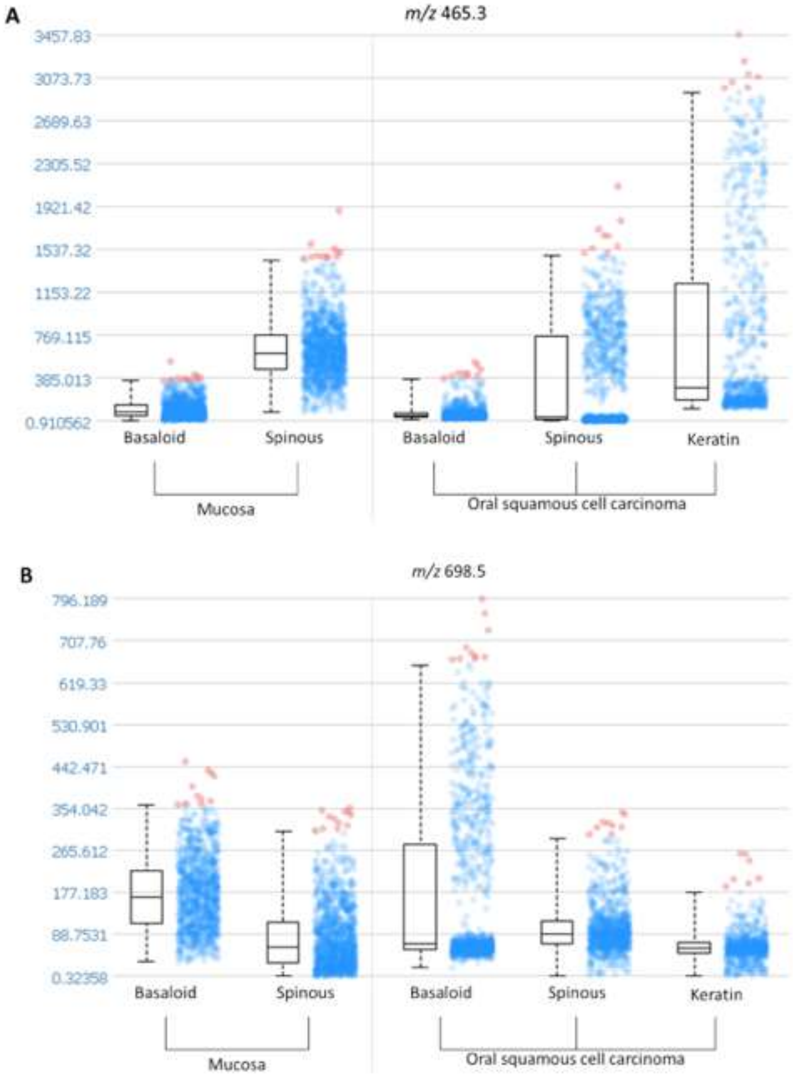

Supplementary Figure S9. Intensity box plots of mass features m/z 465.3 (A) and m/z 698.5 (B) in normal mucosa (basaloid and spinous) and in oral squamous cell carcinoma viable tumor parts (basaloid, spinous, keratin) analyzed by DESI-MSI and normalized on total ion count.

While *m/z* 465.3 intensity tends to increase, *m/z* 698.5 intensity tends to decrease with mucosa and tumor differentiation/keratinization.

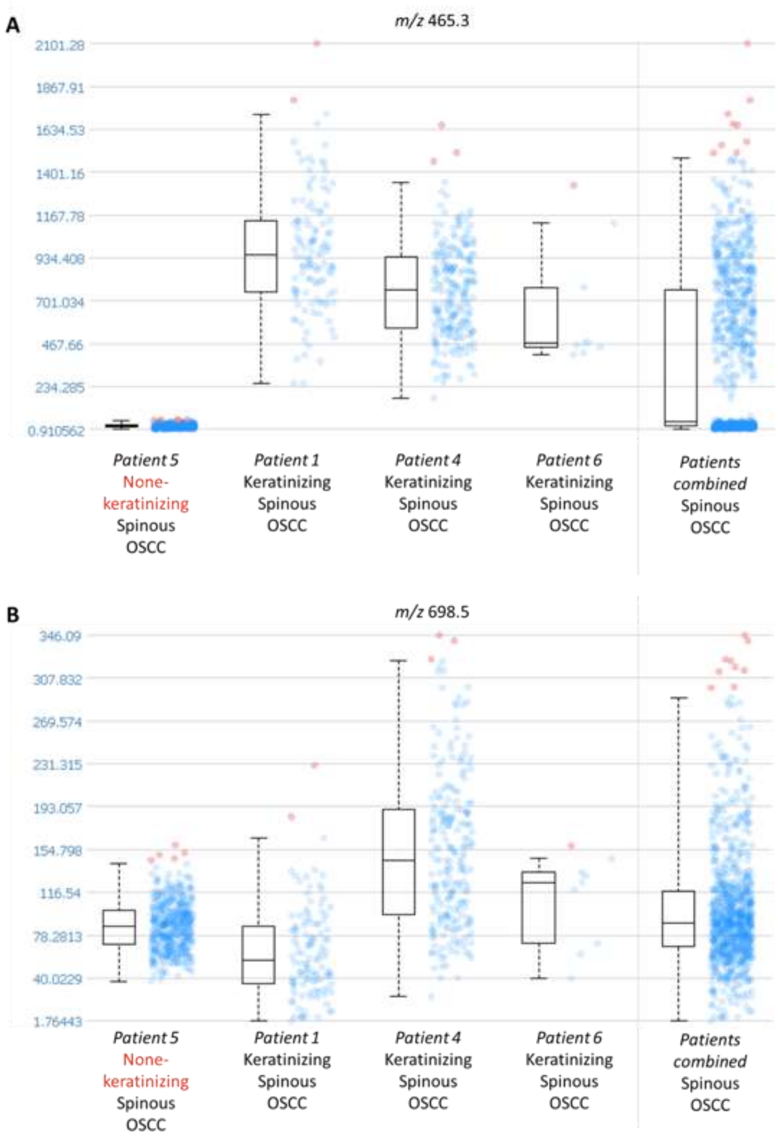

Supplementary Figure S10. Intensity box plots of m/z 465.3 (A) and m/z 698.5 (B) in spinous oral squamous cell carcinoma (OSCC) viable tumor areas analyzed by DESI-MSI, normalized on total ion count for each patient.

While the metabolic profiles of the viable tumor of patients P1, P4 and P6 were extracted from keratinising spinous OSCC, the viable tumor of patient 5 was none-keratinising spinous OSCC and its metabolic profiles present substantially less intense *m/z* 465.3 (A), while *m/z* 698.5 does not show any difference compared to the other keratinizing spinous OSCC.

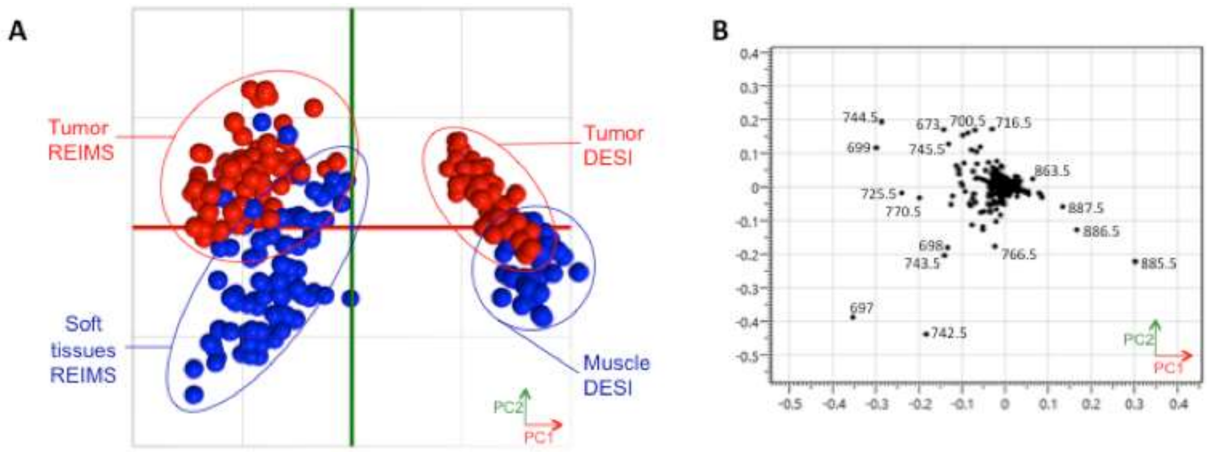

C

Overall class rate		DESI profiles Predicted class by **REIMS classifier**	
50.5%		Soft tissues	Tumor
Actual class	Muscle	100%	0%
	Tumor	100%	0%

D

Overall class rate		REIMS profiles Predicted class by **DESI classifier**	
86.9%		Muscle	Tumor
Actual class	Soft tissues	74.5%	25.6%
	Tumor	0%	97.8%

Supplementary Figure S11. Classifier of REIMS and DESI-MS metabolic profiles.

(A) PCA score plot (PC1 which explains 49.0% of the variance, PC1 12.8%) of 185 REIMS metabolic profiles (94 soft tissue and 91 tumor, used for fig .2) and 107 DESI-MS metabolic profiles (54 muscle and 53 tumor, used for fig.3) on the mass range *m/z* 600-1000.

(B) PC1/PC2 mass features loading plot of the distrubtion of REIMS and DESI-MS metabolic profiles.

(C) Confusion matrix for the classification of 107 DESI-MS metabolic profiles (54 muscle and 53 tumor) with predicted class by REIMS metabolic profiles and actual class defined by histopathology.

(D) Confusion matrix for the classification of the 185 REIMS metabolic profiles (94 soft tissue and 91 tumor) with predicted class by DESI-MS metabolic profiles and actual class defined by histopathology.

REIMS and DESI-MS metabolic profiles were separated along the PC1 axis in the PCA score plot. Tumor and soft tissue/muscle profiles showed a separation along the PC2 axis, despite their belonging to REIMS or DESI-MS metabolic profile classes. REIMS metabolic profiles showed a greater variance than the DESI-MS metabolic profiles. While REIMS classifier was not able to classify properly DESI-MS profiles (i.e. all DESI-MS profiles were predicted as soft tissues), DESI classifier showed an overall accuracy of 86.9%. The greater variance of REIMS metabolic profiles compared to DESI-MS metabolic profiles may explain the poor accuacy of the REIMS classifier. The betterly assessed histopathology of the DESI-MS profiles (i.e. tissue visible and histologically assessed after sampling and betterly defined as muscle) may explain the better accuracy of the DESI classifier.

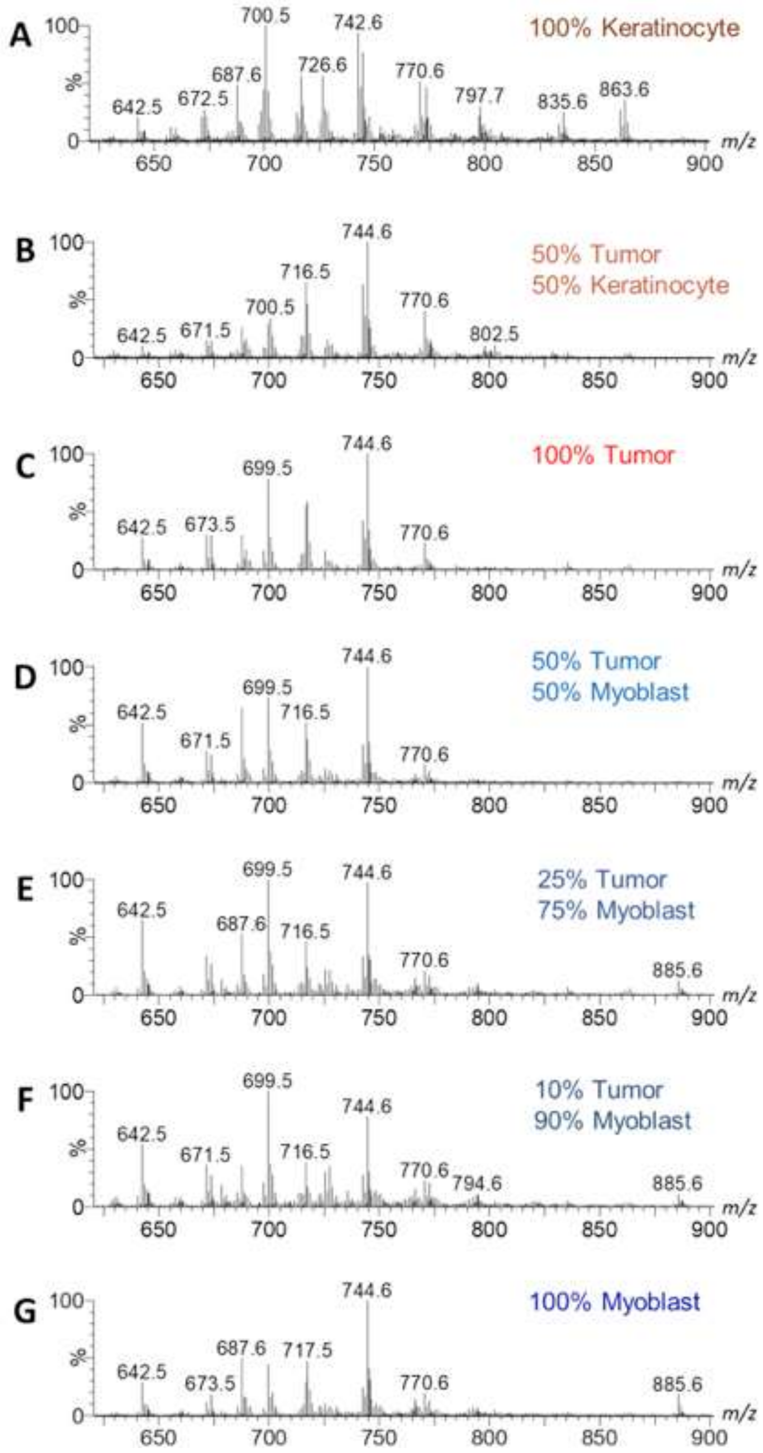

Supplementary Figure S12. REIMS metabolic profiles of cell line mixes.

(A) Pure 100% keratinocyte cells
(B) Mix 50% tumor cells-50% keratinocyte cells
(C) Pure 100% tumor cells
(D) Mix 50% tumor cells-50% myoblast cells
(E) Mix 25% tumor cells-75% myoblast cells
(F) Mix 10% tumor cells-90% myoblast cells
(G) Pure 100% myoblast cells

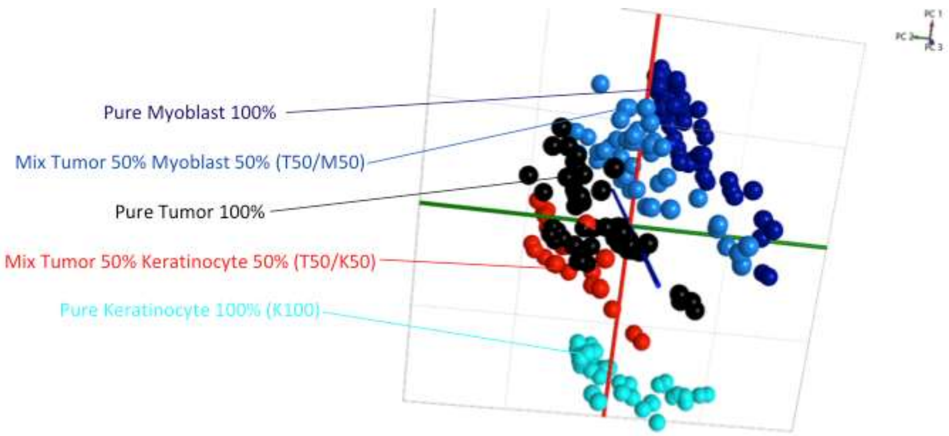

Supplementary Figure S13. PCA score plot related to Fig. 5.

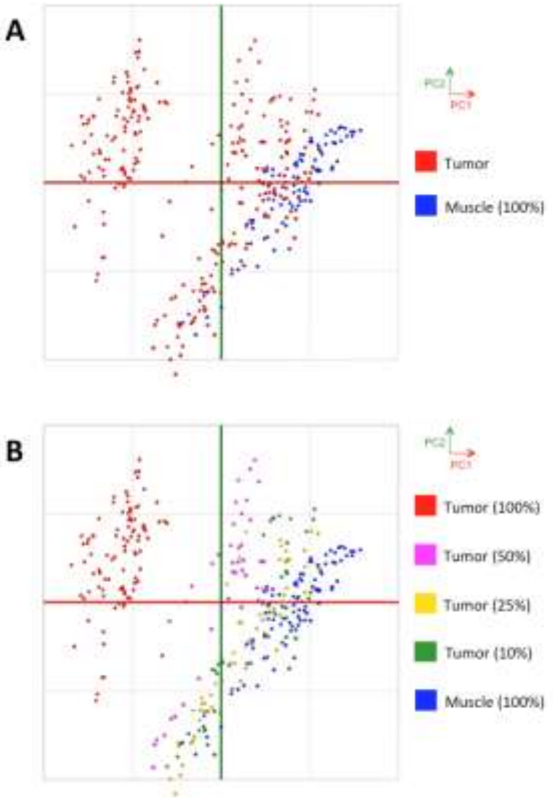

Supplementary Figure S14. (A) PCA score plot related to the model used to calculate the ROC curve. (B) Same score plot but using a different color coding to localize the data with 100% (red), 50% (pink), 25% (yellow), 10% (green) tumor cells and 100% muscle cells.

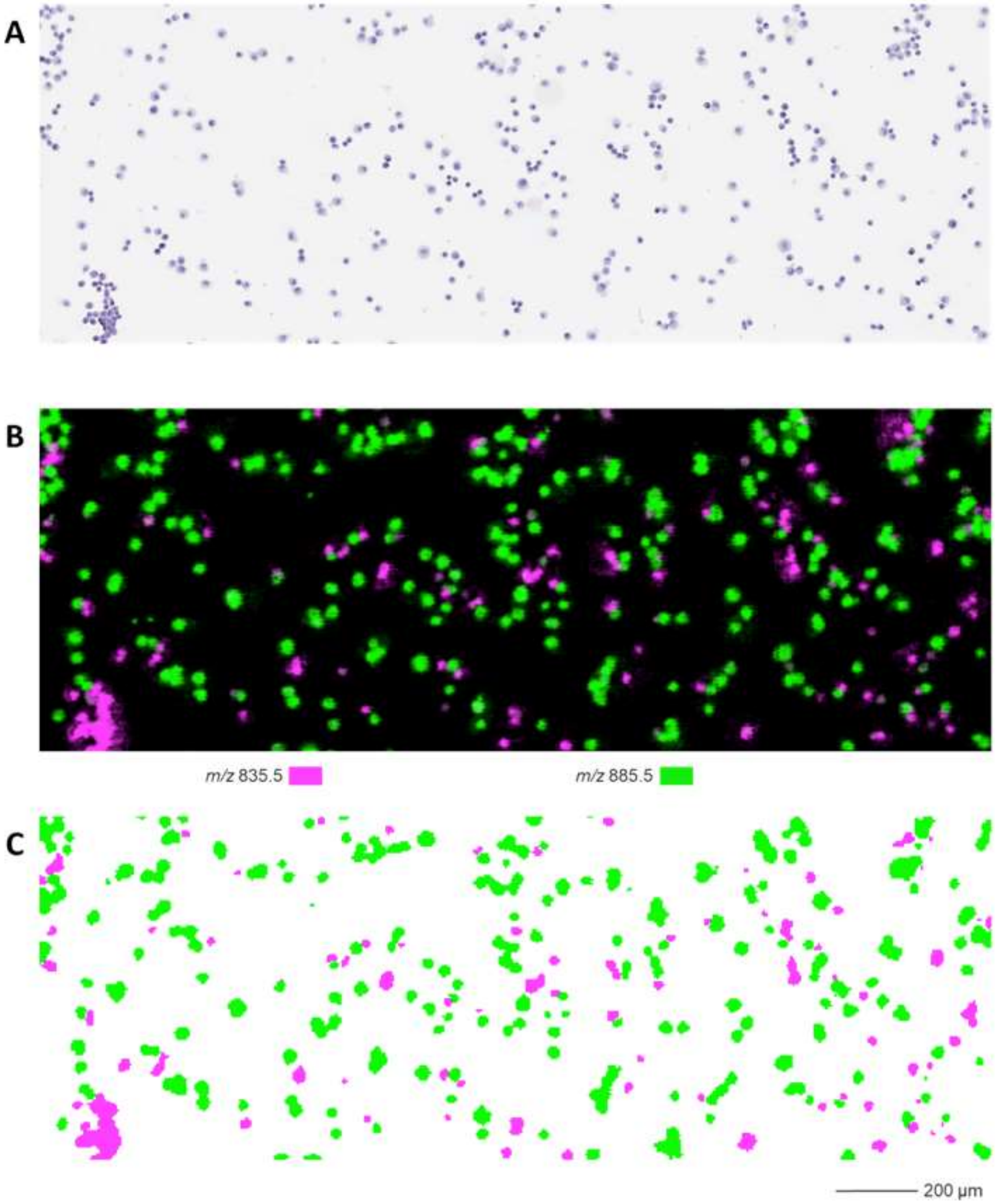

Supplementary Figure S15. Cytospin slide preparation to assess the quality of the cell line mixtures. Example of a mixture tumor/myoblast cells shows clumping of one type of cells on the left bottom corner potentially indicating an partially unhomogenous cell pellet preparation.

(A) Conventional hematoxylin and eosin staining with limited consistency to distinguish two type of cell lines.

(B) Matrix assisted laser-desorption ionization-mass spectrometry imaging (MALDI-MSI) molecular distributions of *m/z* 835.5 and *m/z* 885.5 distinguishing two types of cells.

(C) Segmentation analysis of MALDI-MSI data could check the dilution ratio based on the surface occupied by each type of cells.

References for the supplementary material

(1) Thorley, M.; Duguez, S.; Mazza, E. M. C.; Valsoni, S.; Bigot, A.; Mamchaoui, K.; Harmon, B.; Voit, T.; Mouly, V.; Duddy, W. Skeletal muscle characteristics are preserved in hTERT/cdk4 human myogenic cell lines. *Skelet Muscle* **2016**, *6* (1), 43.
(2) Pauling, J. K.; Hermansson, M.; Hartler, J.; Christiansen, K.; Gallego, S. F.; Peng, B.; Ahrends, R.; Ejsing, C. S. Proposal for a common nomenclature for fragment ions in mass spectra of lipids. *PLOS ONE* **2017**, *12* (11), e0188394.
(3) Guenther, S.; Muirhead, L. J.; Speller, A. V. M.; Golf, O.; Strittmatter, N.; Ramakrishnan, R.; Goldin, R. D.; Jones, E.; Veselkov, K.; Nicholson, J.; et al. Spatially Resolved Metabolic Phenotyping of Breast Cancer by Desorption Electrospray Ionization Mass Spectrometry. *Cancer Research* **2015**, *75*, 1828-1837. St John, E. R.; Balog, J.; McKenzie, J. S.; Rossi, M.; Covington, A.; Muirhead, L.; Bodai, Z.; Rosini, F.; Speller, A. V. M.; Shousha, S.; et al. Rapid evaporative ionisation mass spectrometry of electrosurgical vapours for the identification of breast pathology: towards an intelligent knife for breast cancer surgery. *Breast Cancer Research* **2017**, *19* (1), 59.
(4) Bryne, M.; Koppang, H. S.; Lilleng, R.; Kjærheim, Å. Malignancy grading of the deep invasive margins of oral squamous cell carcinomas has high prognostic value. *The Journal of Pathology* **1992**, *166* (4), 375-381. Brandwein-Gensler, M.; Teixeira, M. S.; Lewis, C. M.; Lee, B.; Rolnitzky, L.; Hille, J. J.; Genden, E.; Urken, M. L.; Wang, B. Y. Oral squamous cell carcinoma: histologic risk assessment, but not margin status, is strongly predictive of local disease-free and overall survival. *The American journal of surgical pathology* **2005**, *29* (2), 167-178. Li, Y.; Bai, S.; Carroll, W.; Dayan, D.; Dort, J. C.; Heller, K.; Jour, G.; Lau, H.; Penner, C.; Prystowsky, M.; et al. Validation of the risk model: high-risk classification and tumor pattern of invasion predict outcome for patients with low-stage oral cavity squamous cell carcinoma. *Head and neck pathology* **2013**, *7* (3), 211-223. Rahman, N.; MacNeill, M.; Wallace, W.; Conn, B. Reframing Histological Risk Assessment of Oral Squamous Cell Carcinoma in the Era of UICC 8th Edition TNM Staging. *Head and neck pathology* **2021**, *15* (1), 202-211.
